# Elevated Cytokine Levels in Plasma of Patients with SARS-CoV-2 Do Not Contribute to Pulmonary Microvascular Endothelial Permeability

**DOI:** 10.1128/spectrum.01671-21

**Published:** 2022-02-16

**Authors:** Anita Kovacs-Kasa, Abdelrahman A. Zaied, Silvia Leanhart, Murat Koseoglu, Supriya Sridhar, Rudolf Lucas, David J. Fulton, Jose A. Vazquez, Brian H. Annex

**Affiliations:** a Vascular Biology Center, Medical College of Georgia at Augusta University, Augusta, Georgia, USA; b Pharmacology and Toxicology, Medical College of Georgia at Augusta University, Augusta, Georgia, USA; c Department of Medicine, Medical College of Georgia at Augusta University, Augusta, Georgia, USA; d Division of Infectious Diseases, Department of Medicine, Medical College of Georgia at Augusta University, Augusta, Georgia, USA; National Institutes of Health

**Keywords:** SARS-CoV-2, barrier dysfunction, endothelial permeability, plasma, cytokine, complements factors, ACE-2 receptor, SARS-CoV-2 plasma, antiinflammatory cytokines, endothelial injury, heat inactivation, neutralizing antibodies, proinflammatory cytokines, spike protein

## Abstract

The vascular endothelial injury occurs in severe acute respiratory syndrome coronavirus 2 (SARS-CoV-2) infections, but the mechanisms are poorly understood. We sought to determine the frequency and type of cytokine elevations and their relationship to endothelial injury induced by plasma from patients with SARS-CoV-2 versus controls. Plasma from eight consecutively enrolled patients hospitalized with acute SARS-CoV-2 infection was compared to controls. Endothelial cell (EC) barrier integrity was evaluated using ECIS (electric cell-substrate impedance sensing) on human lung microvascular EC. Plasma from all SARS-CoV-2 but none from controls decreased transendothelial resistance to a greater degree than that produced by tumor necrosis factor-alpha (TNF-α), the positive control for the assay. Thrombin, angiopoietin 2 (Ang2), and vascular endothelial growth factor (VEGF), complement factor C3a and C5a, and spike protein increased endothelial permeability, but to a lesser extent and a shorter duration when compared to SARS-CoV-2 plasma. Analysis of Ang2, VEGF, and 15 cytokines measured in plasma revealed striking patient-to-patient variability within the SARS-CoV-2 patients. Pretreatment with thrombin inhibitors, single, or combinations of neutralizing antibodies against cytokines, Ca3 and C5a receptor antagonists, or with ACE2 antibody failed to lessen the SARS-CoV-2 plasma-induced EC permeability. The EC barrier destructive effects of plasma from patients with SARS-CoV-2 were susceptible to heat inactivation. Plasma from patients hospitalized with acute SARS-CoV-2 infection uniformly disrupts lung microvascular integrity. No predicted single, or set of, cytokine(s) accounted for the enhanced vascular permeability, although the factor(s) were heat-labile. A still unidentified but potent circulating factor(s) appears to cause the EC disruption in SARS-CoV-2 infected patients.

**IMPORTANCE** Lung vascular endothelial injury in SARS-CoV-2 patients is one of the most important causes of morbidity and mortality and has been linked to more severe complications including acute respiratory distress syndrome (ARDS) and subsequent death due to multiorgan failure. We have demonstrated that in eight consecutive patients with SARS-CoV-2, who were not selected for evidence of endothelial injury, the diluted plasma-induced intense lung microvascular damage, *in vitro*. Known endothelial barrier-disruptive agents and proposed mediators of increased endothelial permeability in SARS-CoV-2, induced changes in permeability that were smaller in magnitude and shorter in duration than plasma from patients with SARS-CoV-2. The effect on endothelial cell permeability of plasma from patients with SARS-CoV-2 was heat-labile. The main plasma factor that causes the increased endothelial permeability remains to be identified. Our study provides a possible approach for future studies to understand the underlying mechanisms leading to vascular injury in SARS-CoV-2 infections.

## INTRODUCTION

To date, over 271 million people have tested positive for severe acute respiratory syndrome coronavirus 2 (SARS-CoV-2), and more than 5.3 million deaths have been attributed to SARS-CoV-2 according to the latest report from the World Health Organization (WHO)(1). The predominant clinical manifestation in patients with SARS-CoV-2 is respiratory distress, with patients presenting with shortness of breath and chest pain, accompanied by chest X-ray or CT scan findings that vary from minimal infiltrates to the hallmark “multifocal bilateral ground glass-opacities” (2). Patients with SARS-CoV-2 infections can also develop a wide range of nonpulmonary symptoms including encephalitis, cranial nerve alterations, and gastrointestinal manifestations (3). Histopathological reports from patients with SARS-CoV-2 infection have shown endothelial injury in many organ systems, which could account for the diffuse edema, marked increases in systemic coagulability, and even multiorgan failure (4). However, the frequency and the cause of endothelial cell (EC) injury associated with SARS-CoV-2 is unknown.

The vascular endothelium is an active organ that is crucial for the regulation of vascular tone, normal barrier function, prevention of hypercoagulability, and the immune response (5). Endothelial injury and vascular endothelialitis are being considered a major contributor of hyper inflammation during SARS-CoV-2 infection (4, 6–9). Messner et al. identified 27 potential plasma factors that are differentially expressed in plasma from early hospitalized patients with SARS-CoV-2, using ultra-high-throughput clinical proteomics (10). Elevated levels of plasma factors were associated with coagulation (e.g., fibrinogen), the complement system, and inflammation related to interleukin (IL)-6 signaling (10). In another study, next-generation plasma profiling identified more than 200 proteins that were differentially expressed in patients with SARS-CoV-2, related to cytokines and immune response (11). According to Ma et al. complement activation is a unique feature of SARS-CoV-2 pathogenesis, which is supported by increased levels of anaphylatoxins (C3a and C5a) in plasma of patients with SARS-CoV-2 (12–14). The plasma components that have been identified in these studies have been linked to impaired vascular function; however, the underlying mechanisms of endothelial injury in SARS-CoV-2 are not completely understood, and we aimed to study the impact of these factors in SARS-CoV-2 plasma-induced endothelial permeability increase.

Thrombin, which is part of the coagulation cascade, is a well-known agent to disrupt endothelial barrier integrity by inducing acute changes in endothelial cell permeability upon redistribution of VE-cadherin in adherent junctions that normally form and stabilize the endothelial monolayer (15, 16). Activation of the complement system, as part of the innate immunity, could contribute to SARS-CoV-2 (13). The components of the complement system, complement component 1 (C1), C3a, and C5a have been extensively studied in SARS-CoV pathogenesis, although its role remained controversial (17). Studies reported that complement factors C3a and C5a were elevated in the blood of patients with SARS-CoV-2 (18–20), and these were also linked to increased endothelial permeability in *in vitro* experiments (21, 22). Moreover, a recent report, during the preparation of our article, showed evidence of the barrier-disruptive effect of SARS-CoV-2 patient plasma that was not due to the SARS-CoV-2 virus (23).

A series of cytokines, particularly those that may target or disrupt the pulmonary vasculature, has been studied in patients with SARS-CoV-2, and increases in IL-1β, IL-6, IL-17, IL-10, tumor necrosis factor-alpha (TNF-α), and interferon-gamma (IFNγ) levels have been reported in patients with SARS-CoV-2 (24, 25). Clinical trials using humanized IL-6, IL-1β, granulocyte-macrophage colony-stimulating factor (GM-CSF), and TNF-α blocking antibodies have been tried in SARS-CoV-2 infections with variable success (26–30). The use of antibody cocktails, targeting different cytokines, would be a way to effective therapy in SARS-CoV-2 according to a recent report (31).

Heat inactivation (56°C for 15 min) of plasma has been used to inactivate the complement system factors in cell culture experiments to avoid complement-mediated cell lysis (32). Heat inactivation also serves as a safety measure to destroy many pathogens, and it can also affect growth factors, cytokines, chemokines, and immunoglobulins (32). According to Ayache et al., levels of several components were decreased in heat-inactivated plasma that can be linked to vascular injury or inflammation, such as macrophage inflammatory protein 1-α and β (MIP-1α, β), macrophage-derived chemokine (MDC), matrix metalloproteinase (MMP)-1, -2, -3, -10, intercellular adhesion molecule-1 (ICAM-1), vascular cell adhesion molecule-1 (VCAM-1), and L-selectin (32–41).

Studying the direct effects of plasma from patients with SARS-CoV-2 in an *ex vivo* model system can help to define the presence and extent of EC injury and the factors responsible for the injury to enable the targeting of known potential mediators of endothelial injury.

## RESULTS

### Patient characteristics.

We analyzed the plasma of eight consecutively enrolled subjects admitted to the Augusta University Medical Center with confirmed SARS-CoV-2 infection, early in the pandemic. All samples were collected within a 2-month period from April to June 2020. The blood samples were drawn within 24 h of admission. In total, six females and two males were enrolled in this study, with ages ranging between 20 and 78 years (Table S1 in the supplemental material). Half of the patients were directly admitted to the Intensive Care Unit. None of the patients were intubated on the day of admission, although two of the patients were intubated during their hospital course. All of the patients recovered and were eventually discharged. Demographics of the control subjects’ plasma are described in the online supplement.

### Plasma from patients with SARS-CoV-2 significantly increased lung microvascular EC permeability.

**FIG 1:**
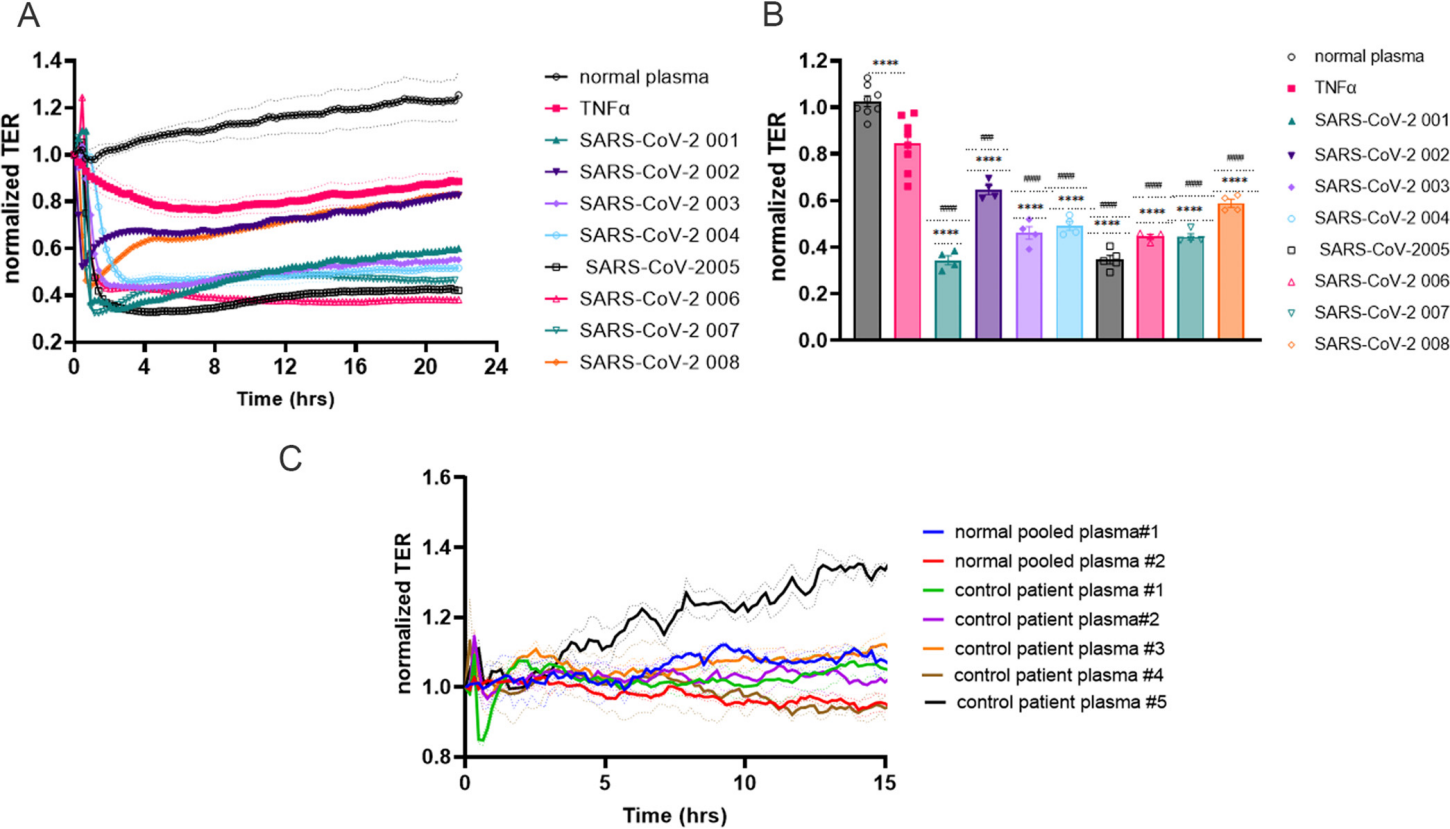
SARS-CoV-2 plasma induces endothelial barrier disruption compared to control plasma. HLMVECs (human lung microvascular endothelial cells) were plated in an ECIS array. Transendothelial electrical resistance (TER) was measured in monolayers over time. The baseline was set, and cells were challenged with either normal or SARS-CoV-2 001–004 (A) patient plasma, or TNF-α (1 ng/mL) at the 0 time point, and TER was monitored for ∼20 h. Bar graphs represent resistance values for normal plasma, TNF-α, or SARS-CoV-2 plasma 001–008 at 2 h after plasma addition. (C) Commercially available pooled plasma or control patient plasma from our biobank was added to the lung EC monolayers, and TER was monitored for 15 h. Normal pooled/control plasma did not induce endothelial permeability increase. Results are expressed as mean ± SEM. Normal plasma and TNF-α are normalized averages from 8 separate experiments. SARS-CoV-2 plasma analyses were done at least 3 times, *n* = 4. The statistical significance was assessed by one‐way ANOVA followed by Bonferroni's multiple comparisons *post hoc* test using Graph Pad Prism. ****, *p* < 0.0001 normal plasma versus TNF-α and SARS-CoV-2 001–008; ####, *p* < 0.0001 TNF-α versus normal plasma and SARS-CoV-2 001–008; ###, *P* = 0.0001.

Real time transendothelial resistance (TER) measurements were performed on human lung microvascular endothelial cells (HLMVEC) using the electrical cell impedance sensing system (ECIS) to evaluate the effect of plasma from the eight subjects enrolled with acute SARS-CoV-2 infection. Plasma from all eight patients with SARS-CoV-2, but none of the five control plasma or the two pooled plasma specimens, caused permeability increases (Fig. 1C). The injury to the endothelial barrier from the highly (1:200 or greater) diluted plasma from the SARS-CoV-2 patients was significantly greater than the tumor necrosis factor alpha (TNF-α) effect, a known EC barrier disruptor (Fig. 1A and B). All of the plasma from patients with SARS-CoV-2 induced a rapid and marked increase in permeability that was present up to 15 h. Of note, we did not detect an increase in permeability when human umbilical vein endothelial cells (HUVECs) were exposed to SARS-CoV-2 plasma compared to plasma from controls or TNF-α (1ng/mL), as shown in Fig. S1.

To confirm that the increased endothelial permeability was not due to cell loss on the plate, we visualized the cells with CD31 marker (Fig. S2A). Interendothelial gap formation and mean fluorescence intensity (MFI) revealed a decreased expression of CD31/area after SARS-CoV-2 plasma exposure for 2 h versus control plasma (Fig. S2B-C). To exclude cell loss, we assessed live cell numbers 1 h after the addition of plasma. None of the plasma specimens from patients with SARS-CoV-2 decreased HLMVEC viability when compared to controls (Fig. S3).

Plasma from patients with SARS-CoV-2 but not plasma from control subjects promoted gap formations in HLMVEC after 1 h of treatment versus controls, as shown by reduced VE-cadherin adherent junction staining that was confirmed by MFI quantification (Fig. S4). Moreover, SARS-CoV-2 plasma promoted F-actin reorganization, indicated by an increased stress fiber formation and a contractile phenotype when compared to control plasma in Fig. S4A. Collectively, SARS-CoV-2 plasma promoted EC barrier disruption, cytoskeleton reorganization, the loss of adherence junctions, and increased gap formation between cells, suggesting a rapid vascular endothelial injury even in highly diluted plasma.

**FIG 2:**
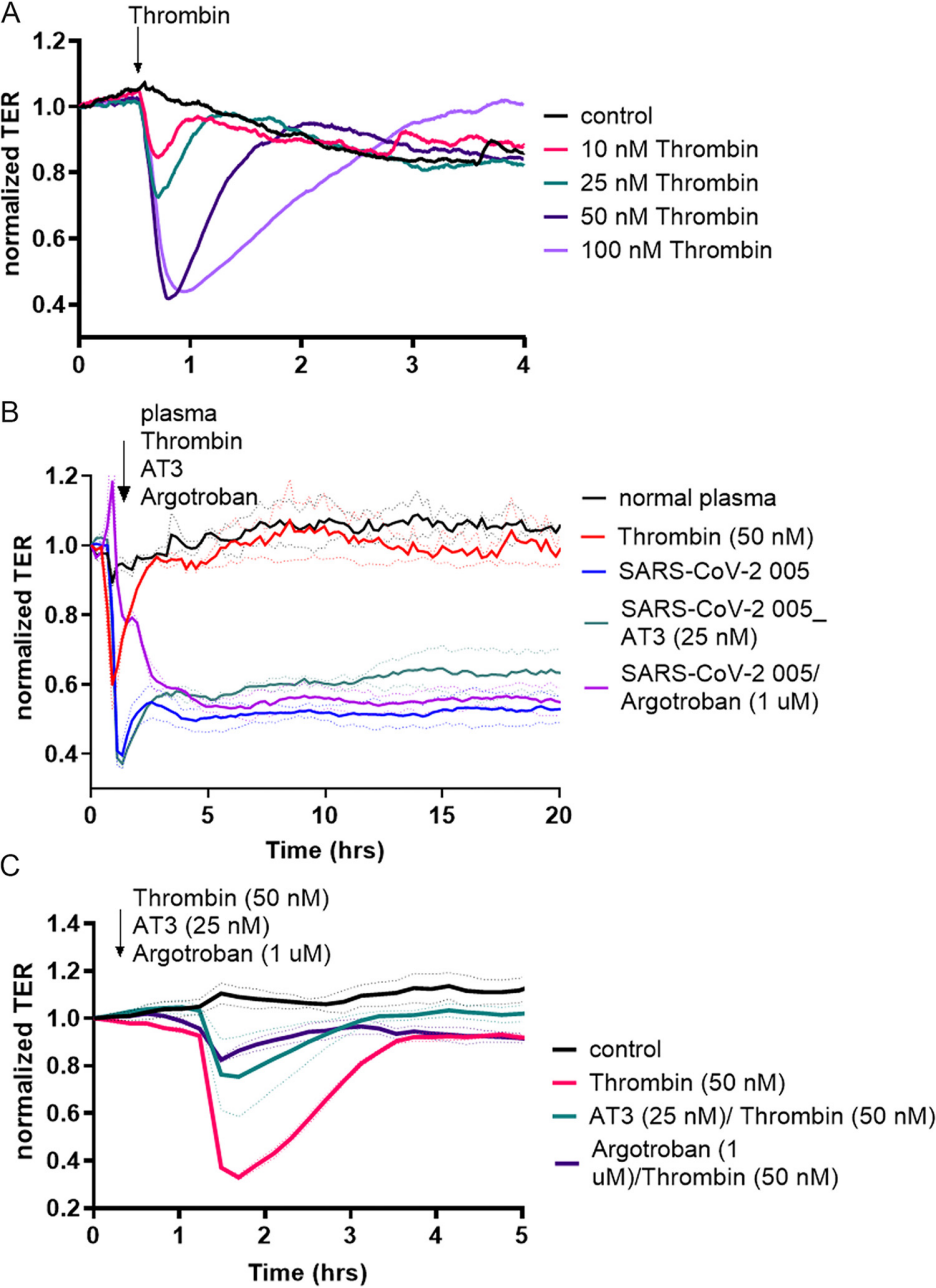
Thrombin inhibitors block thrombin but not SARS-CoV-2 plasma-induced EC permeability increase. HLMVECs (human lung microvascular endothelial cells) were plated in ECIS arrays. Transendothelial electrical resistance (TER) was measured in monolayers over time. The baseline was set, and dose-response analysis was performed with Thrombin. (A) Doses were ranging from 10 nM to 100 nM monitored for 15 h (the first 4 h are shown for better visibility). (B) Thrombin inhibitors, argatroban (1 uM), or antithrombin 3 (AT3, 25 nM) were used to treat the EC monolayers in the presence of SARS-CoV-2 005 patient plasma. (C) Direct thrombin inhibitors (argatroban, 1 uM) or antithrombin 3 (AT3, 25 nM) were used to block thrombin effect on EC permeability. At least 4 independent experiments, *n* = 4.

Thrombin is known to negatively affect endothelial barrier function by disrupting cell–cell connections and promoting gap formation in endothelial monolayer (15, 16). We performed a dose-response analysis with thrombin (10 nM, 25 nM, 50 nM, and 100 nM) and evaluated the effects of thrombin to that of the diluted plasma from patients with SARS-CoV-2 on endothelial permeability (Fig. 2A). The thrombin-induced permeability in microvascular ECs was smaller in magnitude and shorter in duration when compared to SARS-CoV-2 plasma (Fig. 2B). To further exclude thrombin as the main mediator of the increased EC permeability by SARS-CoV-2 plasma treatment, we applied a direct thrombin inhibitor, argatroban (1uM) or antithrombin-3 (AT3, 25 nM), in the presence of thrombin and SARS-CoV-2 plasma (Fig. 2B). Thrombin inhibitors diminished the effect of thrombin but did not alter the SARS-CoV-2 plasma-induced EC permeability increase (Fig. 2B and C).

### Cytokine profiling of plasma from patients with SARS-CoV-2 versus controls.

**FIG 3:**
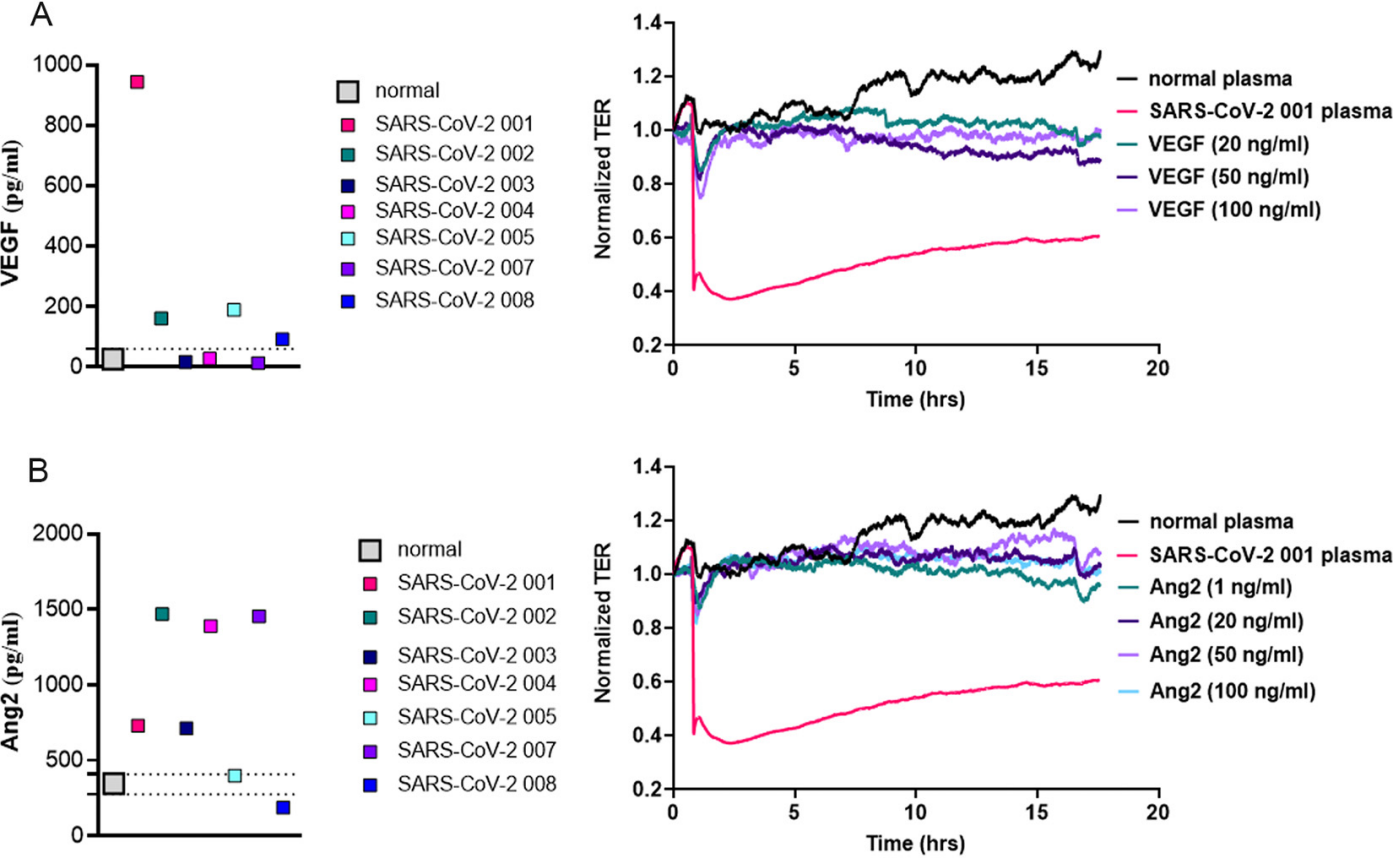
VEGF and Ang2 levels in patient plasma and their effect on EC permeability. (A) VEGF levels in 7 SARS-CoV-2 plasma (001–005, 007, 008) and in normal pooled plasma were determined by the Quantikine Human VEGF Immunoassay. Optical density (OD) values were measured at 450 nm. TER was measured in confluent HLMVEC after the baseline was set. Cells were treated either with normal pooled plasma, withSARS-CoV-2 001 plasma, or with 20, 50, or 100 ng/mL of VEGF. Changes in TER were monitored for 18 h. (B) Ang2 levels in 7 SARS-CoV-2 plasma (001–005, 007, 008) and in normal pooled plasma were analyzed by the Quantikine Human Ang2 Immunoassay. OD values were measured at 450 nm. TER was measured on HLMVEC after baseline was set. Cells were treated either with normal pooled plasma, with SARS-CoV-2 001 plasma, or with 1, 20, 50, or 100 ng/mL of Ang2. The dashed line on the left panels of the figure shows the level of the VEGF or Ang2 levels in the normal plasma samples. Changes in TER were monitored for 18 h.

Vascular endothelial growth factor (VEGF) and angiopoietin 2 (Ang2) are known to be associated with increased EC permeability by TER (42). We analyzed the levels of VEGF and Ang2 in plasma from patients with SARS-CoV-2 and compared it to control plasma by ELISA (Fig. 3). Plasma from all patients with SARS-CoV-2 induced a marked and sustained endothelial injury when compared to control subjects, but VEGF levels when compared to controls were elevated in four of the seven patient samples, lower in one, and no different in two plasma samples (Fig. 3A, left graph). VEGF has been shown to evoke a sustained increase in endothelial permeability at 20–100 ng/mL, whereas lower concentrations (10 ng/mL) have no effect on EC permeability (43). The highest VEGF plasma concentrations in any of the plasma samples from patients with SARS-CoV-2 was ∼1 ng/mL. Moreover, the plasma from patients with SARS-CoV-2 were diluted 1:200, which would result in a final concentration of only ∼5 pg/mL of VEGF in the ECIS assay. Even when recombinant VEGF (20–100 ng/mL) altered EC permeability, the response was not comparable in magnitude nor as sustained as we found in plasma from SARS-CoV-2 patients (Fig. 3A, right panel).

Similarly, when compared to values from plasma from control patients, Ang2 levels in plasma were higher in five of the seven plasma samples from patients with SARS-CoV-2 and were no different than controls in two plasma samples (Fig. 3B, left graph). The maximum Ang2 levels were ∼1.5 ng/mL in SARS-CoV-2 plasma, and with dilution, this would yield levels of ∼7.5 pg/mL, which is much lower than the (1-100 ng/mL) Ang2 needed to increase EC permeability (Fig. 3B, right panel). The Ang2-mediated increases in HLMVEC permeability showed smaller changes and a more rapid recovery even at higher concentrations as compared with SARS-CoV-2 plasma. Moreover, we analyzed the spike-protein (SP) levels in patients with SARS-CoV-2 and found that the SP levels were not detectable in 7 out of 9 patients in diluted plasma from SARS-CoV-2, and only two patients had elevated levels of SP, although the amounts (100 and 120 ng/mL) would not increase EC permeability (Fig. S5). VEGF, Ang2, and the spike protein in plasma were not able to account for the EC permeability increase seen in SARS-CoV-2 patient plasma.

**FIG 4:**
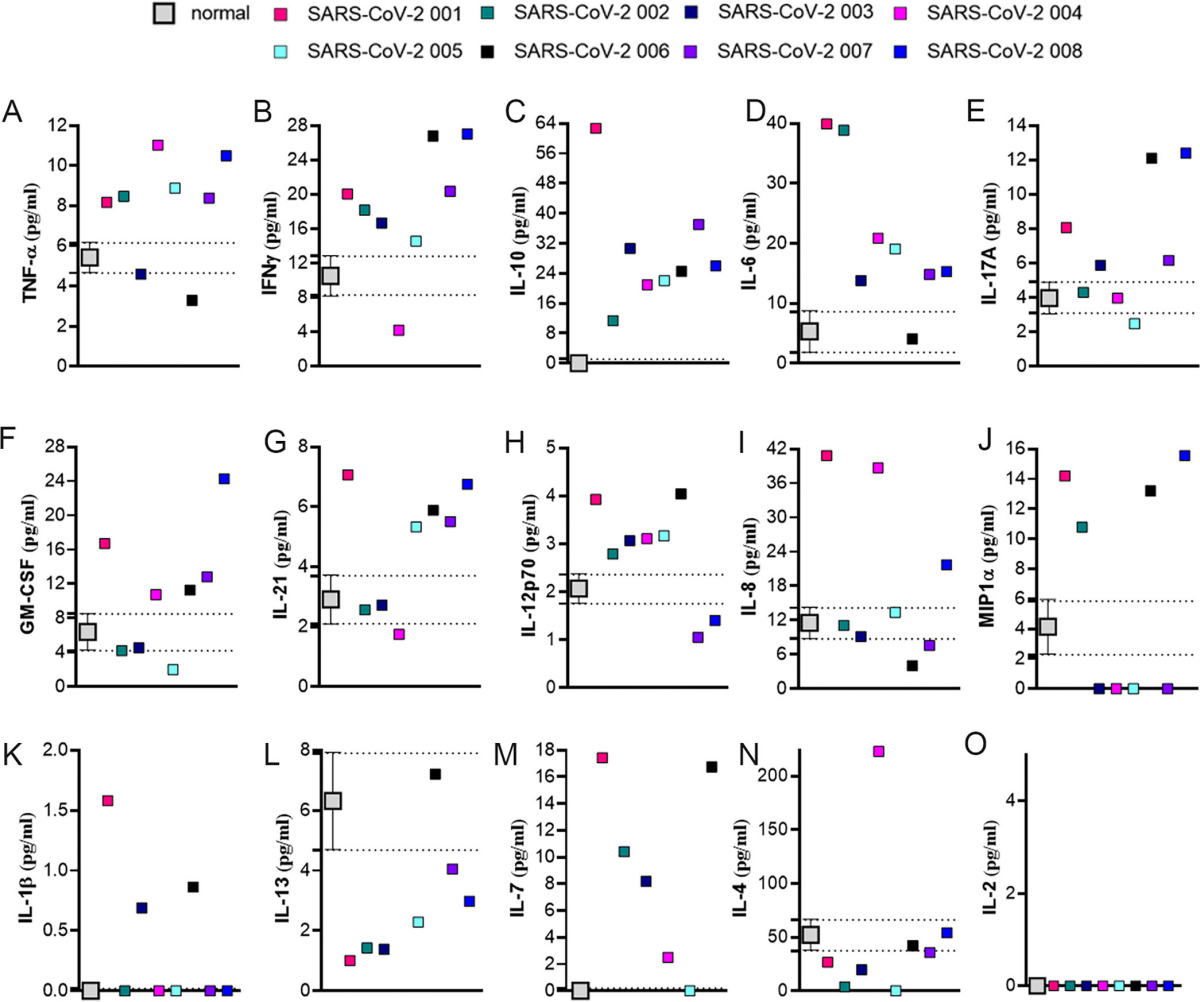
Cytokine profiling of SARS-CoV-2 patient plasma. Inflammatory cytokine levels were analyzed in 8 SARS-CoV-2 patients’ plasma samples in duplicate; 25 μL plasma was analyzed in the assay, using the Milliplex High Sensitivity Human Cytokine Panel (MILLIPLEX MAP– Premixed 15 Plex, EMD Millipore). (A) TNF-α, (B) IFNγ, (C) IL-10, (D) IL-6, (E) IL-17A, (F) GM-CSF, (G) IL-21, (H) IL-12p70, (I) IL-8, (J) MIP1α, (K) IL-1β, (L) IL-13, (M) IL-7, (N) IL-4, (O) IL-2. Commercially available normal pooled plasma was used as the normal plasma group control, shown by mean and SEM. The dashed line shows the level of each cytokine in the normal plasma samples. *n* = 3.

To identify the potential factor(s) in plasma responsible for the increased permeability, we profiled the levels of interleukin (Il)1 beta (IL-1β), IL-2, IL-4, IL-6, IL-7, IL-8, IL-10, IL-12p70, IL-13, IL-17A, IL-21, macrophage inflammatory protein-1 alpha (MIP1α), interferon-gamma (IFNγ), TNF-α, and granulocyte-macrophage colony-stimulating factor (GM-CSF) in seven control and the eight plasma samples from patients with SARS-CoV-2 using the Milliplex High Sensitivity Human Cytokine Panel (44, 45). We found that levels of the proinflammatory cytokines IL-1β, IL-6, IL-7, IL-8, IL-12p70, IL-17A, IL-21, GM-CSF, TNF-α, MIP1α, and IFNγ and antiinflammatory cytokines IL-4, IL-10, and IL-13 were elevated in several of the patients with SARS-CoV-2 (Fig. 4). However, and perhaps more important, we also found significant patient-to-patient variability in the values of these cytokines in plasma from the patients with SARS-CoV-2. For example, TNF-α levels were significantly higher in six out of eight patients’ plasma versus control plasma, while values for two plasma samples from patients with SARS-CoV-2 were similar to control plasma (Fig. 4A). IL-4 levels were decreased in most of the plasma from patients with SARS-CoV-2 when compared to control plasma, except for patient 001(Fig. 4N). IL-2 was not detectable in any of the plasma samples (Fig. 4O). Again, plasma-induced changes in EC permeability versus controls were comparable among all patients with SARS-CoV-2.

### Individual or combinations of neutralizing antibodies failed to attenuate SARS-CoV-2 plasma-induced increase in endothelial permeability.

**FIG 5:**
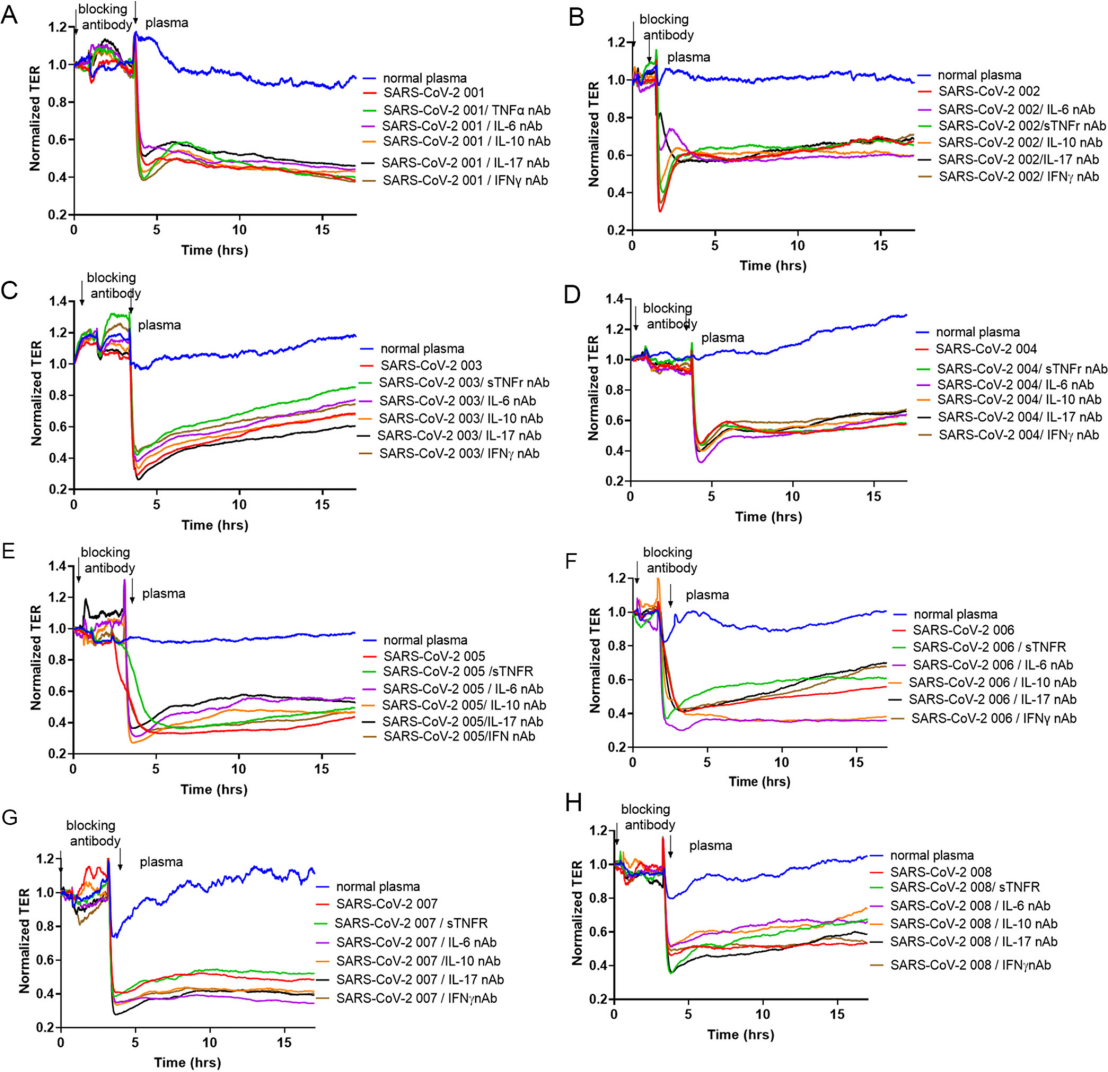
Blocking antibodies to elevated cytokines fail to prevent SARS-CoV-2 plasma mediated increases in EC permeability. Permeability profiling on HLMVEC pretreated for 2 h either with normal plasma, TNF-α (1 ng/mL) blocker, soluble TNF-receptor type I, IL-6 neutralizing antibody (nAb) (5 ng/mL), IL-10 nAb (25 ng/mL), IL-17 nAb (5 ng/mL), or IFNγ nAb (1 ng/mL), then cells were exposed to SARS-CoV-2 plasma (1:200 dilution in normal growth media). Arrows indicate the time point when blocking antibodies and SARS-CoV-2 plasma were added to the cells. TER was monitored for 18 h. Commercially available pooled normal human plasma was used as the normal plasma group control. Results are shown from 3 independent experiments, *n* = 3.

Based on the published effects on endothelial barrier function (Table S2), neutralizing antibodies against IL-6, IL-10, IL-17, TNF-α, and IFNγ were used individually to evaluate the effect on SARS-CoV-2 plasma-induced increases in HLMVEC permeability. However, none of the antibodies had any effect on the endothelial barrier function (Fig. 5). To validate that neutralizing antibodies were able to block the effect of the target cytokines on endothelial permeability, we tested the blocking effect of the IL-17, IFNγ, IL-6, and IL-10 neutralizing antibodies, and the soluble TNF receptor 1 (sTNFr), on IL-17, IFNγ, IL-6, IL-10, and TNF-α-induced endothelial permeability. All neutralizing antibodies were able to block the effect of the target cytokines on endothelial permeability (Fig. S6).

In addition, HLMVEC were pretreated with IL-6, IL-10, IL-17A, or IFNγ blocking antibodies or with sTNFr for 2 h. Afterward, plasma from control subjects or plasma from patients with SARS-CoV-2 was added to the cells, and TER was monitored for 18 h. As shown in Fig. 5, all blocking antibodies failed to reduce the effect of the SARS-CoV-2 plasma on increases in TER in HLMVEC. These results indicate that single cytokine blocking antibody treatments were not able to prevent SARS-CoV-2 plasma-induced disruption of the EC barrier function.

**FIG 6:**
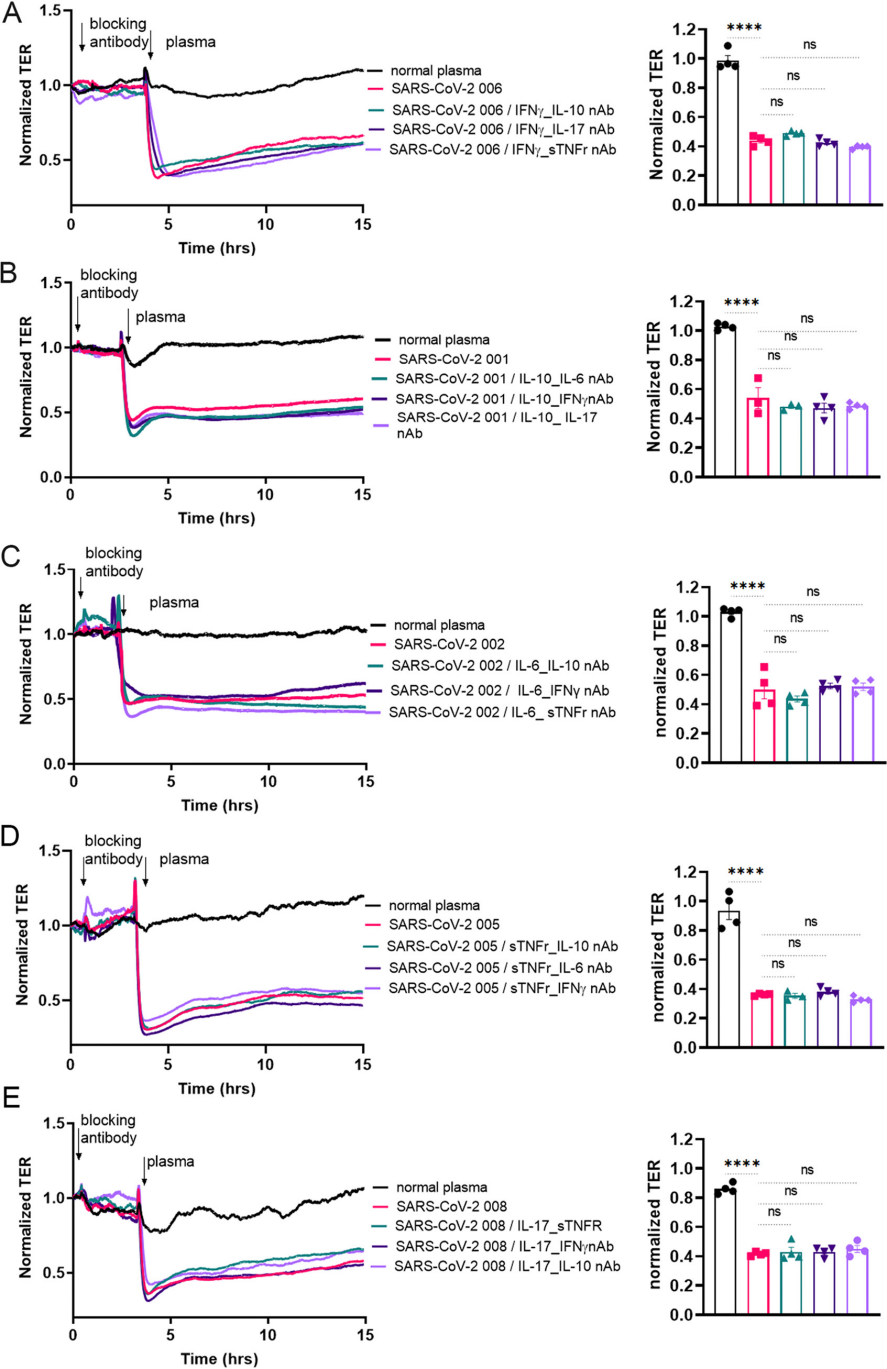
Multiple blocking antibody combinations fail to reduce endothelial injury after exposure to SARS-CoV-2 plasma. HLMVECs monolayers were incubated with combinations of the indicated blocking antibodies for 2 h prior to exposure to SARS-CoV-2 plasma, and TER was monitored for 18 h. For each cytokine target, patient plasma with the highest fold increase is shown: for IFNγ SARS-CoV-2 patient 006, for IL-10 SARS-CoV-2 patient 001, for IL-6 SARS-CoV-2 patient 002, for TNF-α SARS-CoV-2 patient 005, and for IL-17 SARS-CoV-2 patient 008. For each of this selected patient plasma, the three other indicated cytokines with the highest increase were selected as the second target. Arrows indicate the treatment time points. Bar graphs represent the selected time points for statistical analysis to compare each treatment group at 2 h after SARS-CoV-2 plasma treatment. Results are expressed as mean ± SEM of 3 independent experiments, *n* = 5. ****, *P* < 0.0001 normal plasma versus SARS-CoV-2 plasma and SARS-CoV-2 plasma/cytokine combinations. Ns, not significant between groups. The statistical significance was assessed by one‐way ANOVA followed by Tukey’s multiple comparisons *post hoc* test using Graph Pad Prism.

Next, to determine whether a “patient-specific” combination of cytokine neutralizing agents could reduce the effect of SARS-CoV-2 plasma on lung endothelial permeability, blocking antibody combinations were selected based on patient cytokine profiles. HLMVEC were pretreated with combinations of blocking agents against IL-6, IL-10, -IL-17A, IFNγ, or sTNFr for 2 h. Afterward, control or SARS-CoV-2 plasma was added to the cells and TER was monitored for 18 h (Table S3 shows selected antibody combinations in three patients). For the first cytokine neutralizing antibody, the plasma of two patients was selected based on cytokine levels with the highest fold change. An additional third patient’s plasma was used in which the change was the lowest or was not increased at all. For the second cytokine neutralizing antibody, the second, third, and fourth elevated cytokines were selected in each patient. Next, we examined the effect of combining two cytokine blocking antibodies on the ability of SARS-CoV-2 plasma to increase HLMVEC permeability. We found that the combinations of two blocking antibodies against elevated cytokines were also ineffective in protecting the HLMVEC barrier following exposure to SARS-CoV-2 plasma (Fig. 6 and S7). Thus, even patient-specific selected cytokines, when treated with selected blocking agents, did not appear not to be the mediators for the increase in permeability.

### ACE2 blocking antibody failed to attenuate SARS-CoV-2 plasma-induced endothelial permeability.

**FIG 7:**
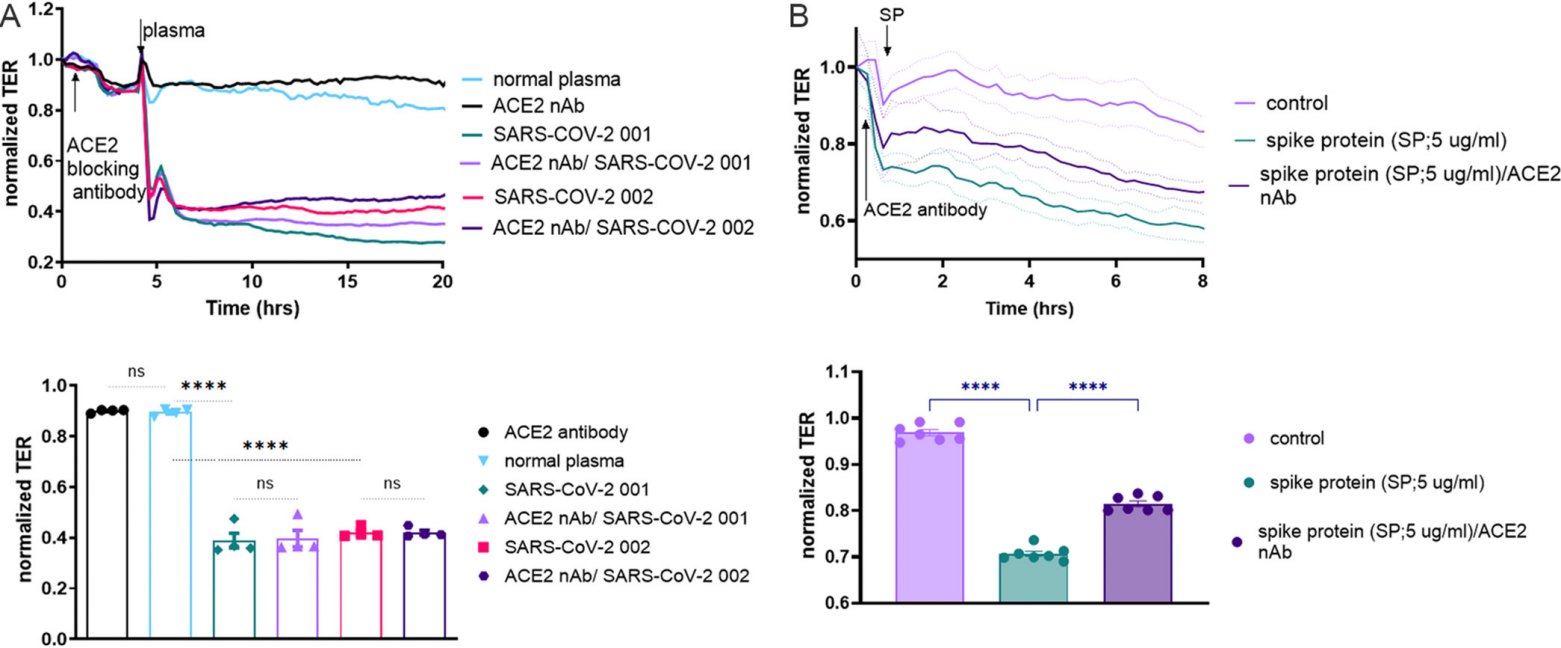
Destruction of the EC barrier by SARS-CoV-2 plasma does not involve ACE2. (A) Barrier function in HLMVEC monolayers was monitored using TER. Cells were exposed to an ACE2 blocking antibody for 2 h after which SARS-CoV-2 plasma from patients 001 and 002 were added (1:200 dilution, in normal growth media) and TER monitored for 18 h. Bar graphs represent the TER values for statistical analysis to compare each treatment group 2 h after SARS-CoV-2 plasma treatment. A one-way ANOVA test was performed to compare three groups, followed by Dunnett’s multiple-comparison test. ****, *P* < 0.0001 normal plasma versus SARS-CoV-2 001/ACE2 nAb, versus SARS-CoV-2 001, versus SARS-CoV-2 002. Ns, not significant SARS-CoV-2 001 versus SARS-CoV-2 001/ACE2 nAb and SARS-CoV-2 002 versus SARS-CoV-2 002/ACE2 nAb. (B) Endothelial monolayers were treated with 5 μg/mL recombinant SARS-CoV-2 spike protein, which increased permeability. ACE2 neutralizing antibody (10 μg/mL) blocked the effect of spike protein on EC permeability. Bar graphs represent the TER values for statistical analysis to compare each treatment group 2 h after SARS-CoV-2 plasma treatment. A one-way ANOVA test was performed to compare three groups, followed by Dunnett’s multiple-comparison test. ****, *P* < 0.0001 control versus spike protein and spike protein versus spike protein/ACE2 nAb. Data represent mean ± SEM, normal plasma. Results are shown from 3 independent experiments, *n* = 4.

To investigate whether SARS-CoV-2, through its spike protein, is directly responsible for the rapid EC barrier disruption, we performed TER measurements using an angiotensin-converting enzyme −2 (ACE 2) blocking antibody, which should prevent binding of SARS-CoV-2 spike protein to human ACE2. HLMVEC were pretreated with the ACE2 antibody for 2 h prior to exposure to SARS-CoV-2 plasma. Afterward, TER was monitored for 18 h. Plasma from patient 001 and patient 002 was tested since each caused a significant increase in EC permeability. However, blocking ACE2 failed to protect from the deterioration of the barrier function induced by the plasma from these patients with SARS-CoV-2 (Fig. 7A). To test the effectiveness of the ACE2 neutralizing antibody, we treated the cells with exogenous spike protein (5 μg/mL, S1, RBD, receptor binding domain) and show that ACE2 antibody treatment (10 μg/μL) blocks the spike protein-induced endothelial permeability increase (Fig. 7B).

### Complement factors C3a and C5a did not contribute to endothelial permeability increase caused by SARS-CoV-2 plasma.

**FIG 8:**
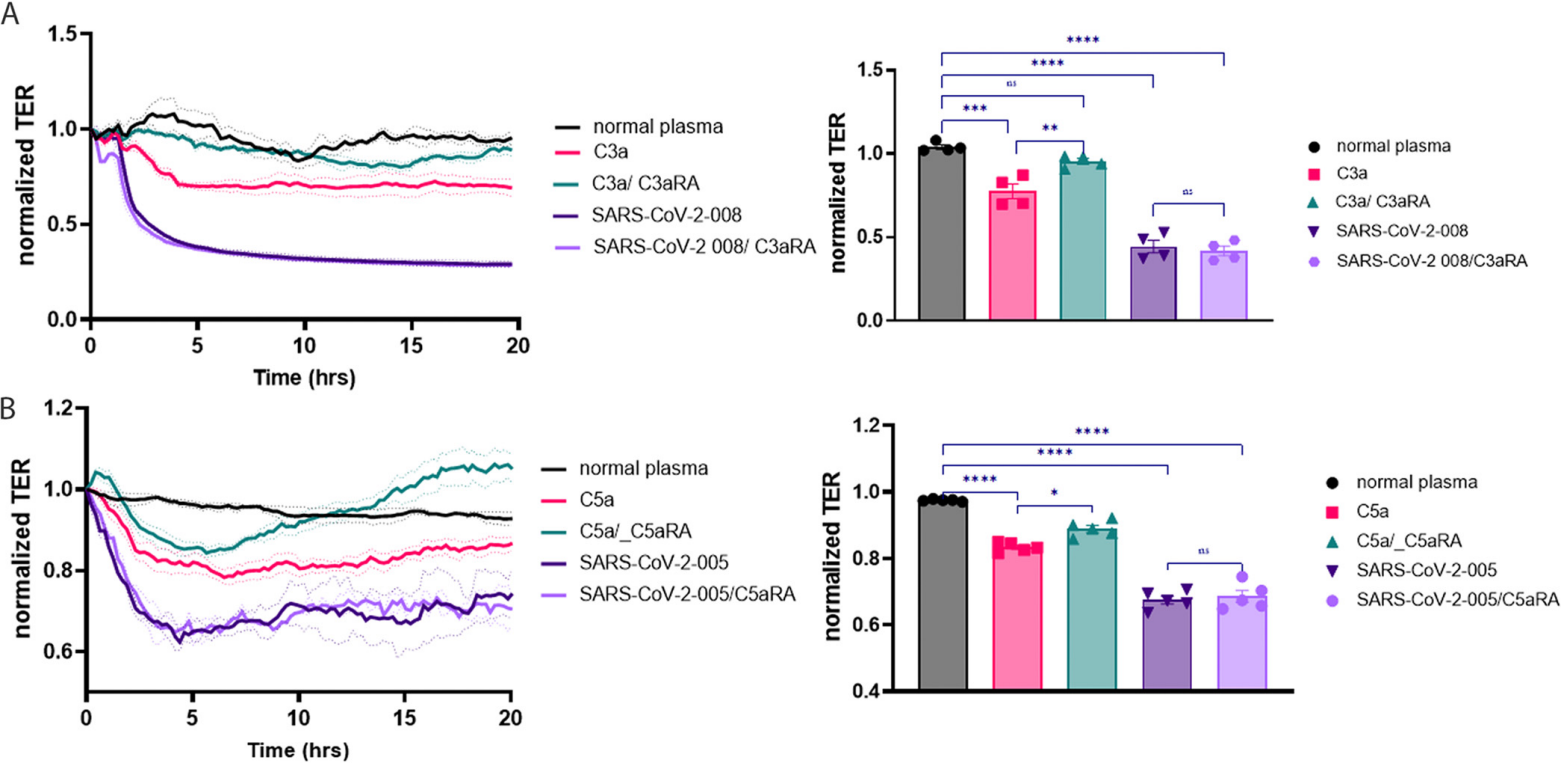
Complement factors C3a and C5a are not responsible for SARS-CoV-2 patient plasma-induced permeability increase. HLMVEC monolayers on ECIS plates were treated either with plasma from the normal patient or plasma from SARS-CoV-2 patients 005 or 008, C3a (A) or C5a (B) with or without the C3a or C5a receptor antagonist (C3aRA, SB290157 100 nM or C5aRA W54011, 100 nM). TER was measured continuously for 20 h. Bar graphs represent the selected time points for statistical analysis to compare each treatment group at 2 h after SARS-CoV-2 plasma treatment. Results are expressed as mean ± SEM of 3 independent experiments, *n* = 3. ****, *P* < 0.0001 versus normal plasma; ***, *P* < 0.001 versus normal plasma; **, *P* < 0.01 C3a versus C3aRA; *, *P* < 0.01 C5a versus C5aRA; ns, SARS-CoV-2 versus SARS-CoV-2 C3aRA or C5Ara. The statistical significance was assessed by one‐way ANOVA followed by Tukey’s multiple comparisons *post hoc* test using Graph Pad Prism.

The complement system is an arm of the innate immune system, playing a role in the immune response against SARS-CoV-2 infection (18). Several components, such as C3a, were detectable in high concentrations in plasma samples of patients with SARS-CoV-2, and C3a levels were correlated with the severity of the disease (19). Since C3a and C5a components have been shown to play a role in microvascular permeability, we examined whether the C3a (Fig. 8A) or C5a (Fig. 8B) receptor antagonist (C3aRA, SB SB290157 100 nM; C5aRA, W54011, 100 nM) could decrease the SARS-CoV-2 plasma-induced EC permeability increase. C3a (100 nM) or C5 (100 nM) treatment decreased TER in lung microvascular ECs (Fig. 8A and B). The treatment with C3aRA or C5aAR blocked the barrier disruptive effect of C3a and C5a but not the effect of the plasma from the patients with SARS-CoV-2 (Fig. 8A and B). In conclusion, the factors, C3a or C5a, involved in complement activation are not responsible for the increased permeability in SARS-CoV-2 infection, as we have not observed SARS-CoV-2-induced permeability change when using the C3a or C5a inhibitors (Fig. 8A and B).

### Heat inactivation of plasma from SARS-CoV-2 patients eliminates barrier disruptive effects.

**FIG 9:**
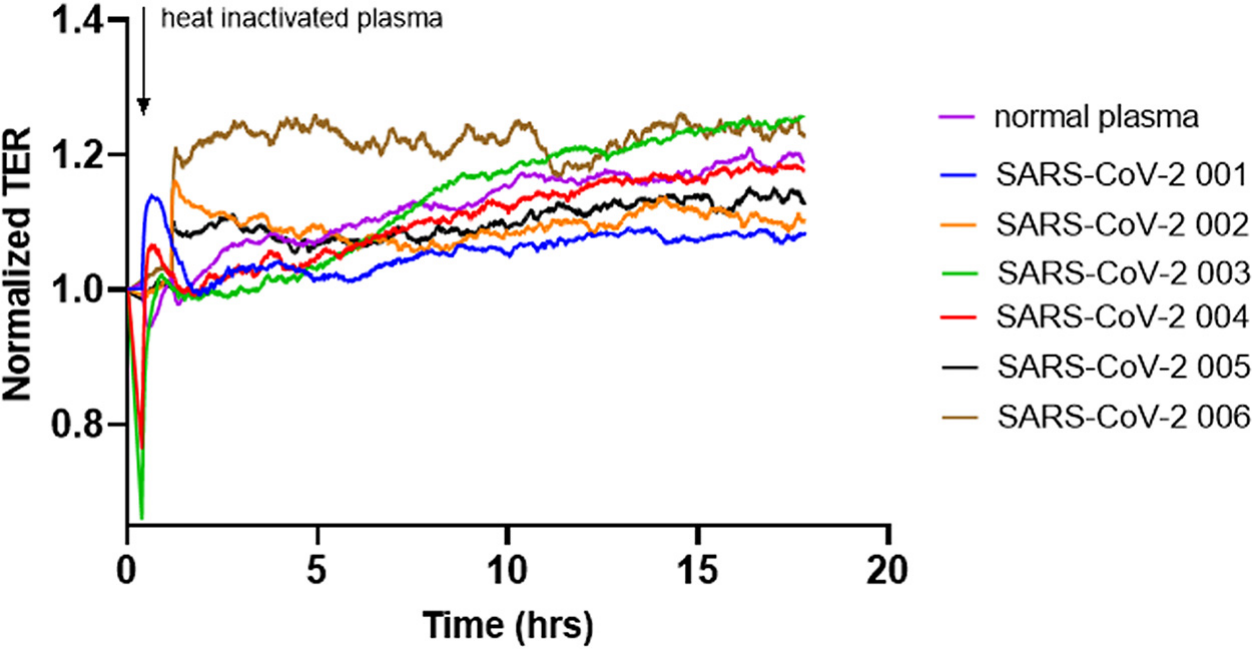
Heat inactivation of SARS-CoV-2 plasma abolishes its barrier disruptive effect. Plasma from SARS-CoV-2 patients (001–006) was heat-inactivated at 56°C for 15 min and applied to HLMVEC, and TER was measured for 18 h. Arrow indicates the time point when the plasma was added to the cells. Results are shown from 3 independent experiments, *n* = 4.

Heat-inactivation was evaluated as a method of eliminating the barrier disruption function of SARS-CoV-2 plasma. Heat inactivation was achieved by incubating plasma samples from SARS-CoV-2 patients at 56°C for 15 min (46). Heat inactivation completely prevented the ability of the plasma to increase endothelial permeability (Fig. 9). Collectively, these data indicate that the plasma component responsible for compromising EC permeability appears to be a heat-labile protein.

## DISCUSSION

In the present study, we have demonstrated that plasma from patients with acute SARS-CoV-2 infection, even at a (1:200) dilution, evokes a pronounced and prolonged increase in human lung microvascular endothelial permeability. Michalick et al. have recently reported the EC barrier disrupting effects of SARS-CoV-2 plasma on lung ECs (23). Although our study demonstrates some similar results and thus supports their data, we have been able to expand the information by examining circulating cytokines and showing that tested putative cytokines are not correlated with the increase in EC permeability; targeted blocking antibody studies fail to limit EC barrier disruption, and spike protein is not the cause of permeability increase, but the effect is heat-inactivated. Our results also support the finding of Michalick et al. that the permeability increase from patients’ plasma was not a consequence of SARS-CoV-2, as they did not detect viral RNA in the plasma and we excluded the spike protein (10, 23). The fact that we could not pin-point a single factor is perhaps not surprising as high throughput proteomic analysis showed that 27 proteins, and next generation plasma profiling identified that more than 200 proteins, were elevated in SARS-Cov-2 plasma versus controls representing possible markers of the disease (10, 11). To date, an exact plasma factor responsible for the lung microvascular injury and the robust endothelial permeability increase observed has yet to be fully identified.

In our study, all of the SARS-CoV-2 patient plasma samples demonstrated a significant increase in the degree of EC permeability, which was greater than observed with TNF-α, the positive control for this assay. Thrombin is a well-known endothelial barrier disrupting agent and we tested across doses of thrombin (ranging from 10 nM to 100 nM) versus SARS-CoV-2 plasma to compare their effect on EC permeability increases (16, 47). Our data demonstrate that even 100 nM thrombin was less potent than SARS-CoV-2 plasma in causing increases in microvascular permeability. Moreover, EC monolayers recovered 1–2 h after the addition of thrombin, but not after the addition of the SARS-CoV-2 patient plasma. The direct thrombin inhibitor, argatroban, and the indirect inhibitor, antithrombin 3, blocked the thrombin-mediated EC permeability increases, although they were not as effective in reducing the barrier disruptive effect of SARS-CoV-2 plasma (Fig. 2). We also tested factors such as VEGF and Ang2, which are known to cause an increase in the endothelial permeability, and the postdilution levels of VEGF and Ang2 in plasma were too low to be the factor inducing abnormalities in vascular permeability. While high doses of recombinant VEGF and Ang2 were able to promote EC permeability, the magnitude and duration of injury were less when compared to the effect of the SARS-CoV-2 plasma. Patients with SARS-CoV-2, 003, 004, and 007, did not have elevated VEGF, while patients 005 and 008 did not have elevated Ang2 levels, yet samples caused similar increases in vascular permeability (Fig. 2A). Thus, we can exclude VEGF, Ang2, and thrombin as being solely implicated as potential causative agents that increase endothelial permeability in SARS-CoV-2 (42, 48).

After evaluating the levels of cytokines, we found differences between the SARS-CoV-2 plasma versus controls. However, within the patients with SARS-CoV-2, there was a high degree of patient- to-patient variability (Fig. 3). Prior studies have shown that plasma levels of the proinflammatory cytokines, IL-1β, IL-2, IL-6, IL-8, and IL-17, are increased in patients with SARS-CoV-2 (49–51). Several other investigators have reported elevated levels of TNF-α, IFNγ, IL-7, GM-CSF, and MIP1α, while higher levels of the antiinflammatory IL-4 and IL-10 cytokines would presumably mitigate the hyperinflammatory state (2, 49). Increased IL-6 and IL-10 levels have been reported to be associated with more severe infection due to SARS-CoV-2 (2, 50). Clinical trials have evaluated the effects of the humanized IL-6 inhibitor, tocilizumab, in patients with SARS-CoV-2 and have shown some benefit in morbidity and mortality (26, 27, 52). While variability in cytokine levels could help explain these results, in our study we found that even in patients with elevated IL-6 levels, blocking this cytokine did not attenuate the ability of SARS-CoV-2 plasma to induce endothelial barrier disruption. Studies using neutralizing antibodies to IL-17 or TNF-α in mice and in several clinical trials for acute respiratory distress syndrome (ARDS) demonstrated a positive response (53, 54). In this study, we found that none of the cytokine neutralizing antibodies alone (IL-6, IL-10, IL-17, IFNγ, or soluble TNF receptor I) or in combination were able to reduce the effect of SARS-CoV-2 plasma to increase endothelial permeability.

An unexpected finding of our study was that the antiinflammatory cytokine, IL-10, appeared to be the most consistently elevated among the 15 cytokines studied in plasma from SARS-CoV-2 versus control patients. Another research group has also reported elevated levels of IL-10 in early-stage SARS-CoV-2 patients (2, 55). Although IL-10 is widely regarded as a “protective” antiinflammatory cytokine, there are recent studies suggesting that its increase in severe SARS-CoV-2 infection may contribute to immune cell activation and inflammation (56). Our data should not be taken to suggest that IL-10 blockade be considered as a therapeutic approach for SARS-CoV-2.

Because of the predilection of SARS-CoV-2 for the respiratory tract and the common end result of respiratory failure, we will comment on our results in the context of lung vascular barrier studies and ARDS (57, 58). A major contributor to the pathology of ARDS is an injury to the pulmonary vascular barrier, leading to increased permeability, pulmonary edema, and subsequent hypoxia (33, 34). To date, there is scant information in the literature about whether plasma from patients with ARDS can affect lung microvascular endothelial permeability. One study reported that plasma from patients with ARDS can promote endothelial permeability in HUVECs. However, this involved a brief period of plasma exposure and resulted in a small degree of barrier disruption (35). In our study, we analyzed plasma collected on the first day of admission from enrolled subjects with acute SARS-CoV-2 infection. Attempts were made to consecutively enroll, and these patients were not selected based on the presence of respiratory failure or ARDS. In fact, most subjects did not require intensive care or mechanical ventilation on the first day of enrollment and sample collection. Nevertheless, all of the plasma collected from patients with SARS-CoV-2 showed a significant ability to disrupt the pulmonary microvascular endothelial cell barrier.

In our study, heat inactivation completely blocked the endothelial permeability increase caused by the plasma of patients with SARS-CoV-2 (Fig. 9), which suggests that a heat-labile plasma factor is responsible for the barrier disruptive effect of SARS-CoV-2 plasma. Ayache et al. looked at heat-inactivation of plasma isolated from healthy subjects. In a screen of 100 proteins, a full 1/3 was altered either at the protein or activity level. With plasma containing roughly 300 proteins, this does not significantly reduce the number of candidates (32, 36). Heat inactivation decreased the level of several chemokines, such as macrophage inflammatory protein 1-α and β (MIP-1α, β) and MDC (macrophage-derived chemokine), which are linked to inflammatory processes in lung injury (32, 33). Matrix metalloproteinase (MMP)-1, -2, -3, and -10 levels were also decreased after heat inactivation. MMPs are involved in vascular complications and are associated with endothelial dysfunction (34–36). The levels of intercellular adhesion molecule-1 (ICAM-1), vascular cell adhesion molecule-1 (VCAM-1), and L-selectin were also decreased in heat-inactivated plasma. These adhesion proteins are known to be associated with vascular inflammation (15).

In totality, most of the plasma factors that were affected by heat inactivation can be linked to inflammatory pathways and vascular permeability. We hypothesize that these factors, whose levels were decreased by heat inactivation in the plasma, could account for the increased permeability caused by plasma from patients with SARS-CoV-2 since we found that heat-inactivated SARS-CoV-2 plasma did not increase EC permeability. In our follow-up studies, we will determine the plasma components increased in SARS-Cov-2 and then identify those that are heat-inactivated and are accountable for SARS-CoV-2 induced endothelial permeability increase.

Collectively, our study provides evidence for the disruptive effect of circulating SARS-CoV-2 plasma factors on the vascular endothelium, an effect that could not be accounted for after evaluating a large number of putative, potentially causative factors. It is important to note that the effects on increased vascular permeability were eliminated by heat inactivation, but not by thrombin inhibitors, neutralization of ACE2, VEGF, Ang2, or multiple cytokine blockers. Our findings provide a novel perspective for future studies to identify the mechanisms and signaling pathways involved in SARS-CoV-2 induced endothelial dysfunction and the possibility of developing adjuvant therapies that would prevent the endothelial dysfunction in SARS-CoV-2 infections.

## MATERIALS AND METHODS

Extended methods are available in the online supplemental material.

### Study subjects.

SARS-CoV-2 Shedding, Sequence variation and Immune responses in confirmed and suspected SARS-CoV-2 patients (SSIC Study) was approved by the Institutional Review Board of Augusta University.

### Cell culture.

Human Lung Microvascular ECs (HLMVECs) or human umbilical vein endothelial cells (HUVECs) (Lonza, Morristown, NJ) were used in our study.

### Endothelial permeability measurement with the Electrical Cell Impedance Sensing System (ECIS).

HLMVECs were seeded on gold microelectrodes in eight-well ECIS plates (Applied Biophysics, Troy, NY) as described previously (59).

### Immunostaining.

Immunostaining was performed to visualize CD31, VE-Cadherin, and F-actin in HLMVEC after SARS-CoV-2 patient’s or control plasma treatment (1 h), similar to methods previously described (59). The gap area on an endothelial monolayer of CD31, VE-cadherin, and F-actin was analyzed using ImageJ & Fiji software.

### Cytokine assay.

A panel of 15 cytokines was assessed in plasma from the study subjects, using a highly sensitive cytokine bead assay (MILLIPLEX MAP High Sensitivity Human Cytokine Panel), EMD Millipore (44).

### ELISA.

Levels of VEGF, Ang2, and spike protein were measured from SARS-CoV-2 patients’ samples according to the manufacturer’s recommendations.

### Cell viability.

Human lung microvascular ECs were exposed for 1 h to either control plasma or SARS-CoV-2 patients’ plasma. Afterwards, water-soluble tetrazolium salt, WST-8, was added to the cells for 1 h. Cell viability was assessed by reading the absorbance at 450 nm.

### Statistical analysis.

Results are presented as mean ± SEM for 3–5 experiments. Normality was evaluated using the Shapiro–Wilk test and statistical significance assessed by one‐way ANOVA followed by Tukey’s or Dunnett's multiple comparisons *post hoc* test using Graph Pad Prism. Differences were considered statistically significant at *P* values ≤ 0.05.
