# Supplementary material for: Elevated Cytokine Levels in Plasma of Patients with SARS-CoV-2 Do Not Contribute to Pulmonary Microvascular Endothelial Permeability
Source: Microbiol Spectr. 2022 Feb 16;10(1):e01671-21. doi: 10.1128/spectrum.01671-21 (PMC8849075; doi:10.1128/spectrum.01671-21)
Supplement: SUPPLEMENTAL FILE 1 — Supplemental material. Download SPECTRUM01671-21_Supp_1_seq14.pdf, PDF file, 2.7 MB [file spectrum01671-21_supp_1_seq14.pdf]

ONLINE SUPPLEMENT

Elevated Cytokine Levels in Plasma of Patients with SARS-CoV-2 do not contribute to Pulmonary Microvascular Endothelial Permeability

Authors: Anita Kovacs-Kasa, Abdelrahman Zaied, Silvia Leanhart, Murat Koseoglu, Supriya Sridhar, Rudolf Lucas, David J. Fulton, Jose A. Vazquez, and Brian H. Annex

Methods
Online Figure Legends
Online Figures
References 1-14

METHODS

Data Availability. The authors declare that all supporting data are available within the manuscript and in the Online Supplement.

Study Population

SARS-CoV-2 Shedding, Sequence variation, and Immune responses in confirmed and suspected SARS-CoV-2 patients (SSIC Study) were approved at Augusta University Medical Center. EDTA blood was collected from 8 consecutively enrolled confirmed SARS-CoV-2 positive patients on the day of admission. Whole blood samples were kept on ice and plasma was separated by centrifugation at 2,000 × g for 5 min at 4°C (1). Plasma aliquots were stored at −80°C. In the normal plasma group, we utilized commercially available pooled EDTA plasma (Innovative Research, Novi, MI) and control human EDTA plasma aliquots from our plasma biobank to establish a value range for the controls we used (2). 5 control plasma was used for the ECIS experiments and for the cytokine profiling experiment besides the pooled plasma. Serum was isolated from blood collected in serum separator tubes and stored at -80°C. Normal human serum was purchased from Sigma.

Cell culture

Human Lung Microvascular ECs (HLMVECs) or human umbilical vein endothelial cells (HUVECs) (Lonza, Morristown, NJ) were maintained in EBM^{TM}-2 Basal Medium supplemented with and EGM^{TM}-2 MV Microvascular EC Growth Medium Single Quots^{TM}. Cells were grown at 37°C in a 5% CO_2 incubator and used from passage 2–6.

Endothelial permeability measurement with Electrical Cell Impedance Sensing System (ECIS)

HLMVECs were seeded on gold microelectrodes in 8 well ECIS plates (Applied Biophysics, Troy, NY) as it was described previously (3). Briefly, EC monolayers resistance was tested by measuring transendothelial resistance (TER) in real-time. After the baseline was set, cells were challenged either with normal pooled plasma or SARS-CoV-2 patient's plasma (1:200) in EGM-2, and TER was monitored for 15-20 hours in the presence of plasma or other treatments. IL-6, IL-10, IL-17, IFN-γ, ACE-2 neutralizing antibodies, soluble TNF receptor were used as a 2h pretreatment before plasma treatment. Recombinant SARS-CoV-2 Spike Protein, S1 Subunit, and Host Cell Receptor Binding Domain (RBD) were purchased from Ray Biotech (Atlanta, GA).

Immunostaining

Human Lung ECs were grown to confluence on glass coverslips. HLMVEC were treated with plasma from SARS-CoV-2 patients for 1 hr. Then EC monolayers were washed with 1xPBS twice, then fixed with 3.7% paraformaldehyde (PFA) for 10 minutes on RT. After 5 min RT permeabilization (0.25% Triton X-100 in Tris-buffered saline with 0.1% Tween) HLMVECs were blocked with 2% bovine serum albumin in TBST for 1hr. Primary antibodies were added overnight 1:500 dilution. Secondary antibody in 1:300 was incubated with the cells for 1 hr. between each step, cells were rinsed three times with 1 × PBS. Coverslips were rinsed with 1xPBS, then mounted with ProLong® Gold Antifade Reagent and observed with ×40 immersion oil objective lenses using a Zeiss 780 Upright Confocal microscope at Augusta University Core Lab.

Image Analysis

The Gap area on an endothelial monolayer of CD31, VE-Cadherin, and F-actin was analyzed using ImageJ & Fiji software. The area of interendothelial gaps was outlined manually. The values were expressed as a percentile of the total surface area. Mean fluorescence intensity was analyzed by

ZEN Blue software by selecting the same size area on the normal plasma or SARS-CoV-2 plasma-treated images. Images were thresholded by subtracting the background.

Cytokine assay

A panel of 15 pro-and anti-inflammatory cytokines; interleukin (Il)1 beta (IL-1β), IL-2, IL-4, IL-6, IL-7, IL-8, IL-10, IL-12p70, IL-13, IL-17A, IL-21, macrophage inflammatory protein-1 alpha (MIP1α), interferon-gamma (IFNγ), TNF-α, and granulocyte-macrophage colony-stimulating factor (GM-CSF) was assessed in duplicates in 50 μL plasma from the study subjects, using a highly sensitive cytokine bead assay (MILLIPLEX MAP High Sensitivity Human Cytokine Panel – Premixed 13 Plex, EMD Millipore (4, 5).

ELISA

Human VEGF or Ang2 Quantikine ELISA Kit (R&D Systems, Minneapolis, MN) or COVID-19, Spike Protein ELISA Kit (Abcam Cambridge, MA) were used to measure levels of VEGF and Ang2 in SARS-CoV-2 patient plasma or levels of spike protein in SARS-CoV-2 patients' sera. In total, 100 μL of each standard and sample (1:20) were added into appropriate wells and incubated for 2.5 hours at room temperature. After discarding the solution the plate was washed 4 times with 1X Wash Solution. 100 μL of 1X Biotinylated Angiopoietin 2 Detection Antibody was added to each well, then incubated for 1 hour at RT. After washing 100 ul of 1X HRP-Streptavidin solution was added to the wells and incubated for 45 minutes at RT. 100 μL of TMB One-Step Substrate Reagent was added to the wells for 30 minutes at RT in the dark. After the addition of 50 μL of Stop Solution, absorbance was read at 450 nm.

Cell viability

Human lung microvascular ECs plated in 96 well plate were either treated with normal pooled plasma (1:200 dilution) or SARS-CoV-2 patients' plasma (1:200 dilution) for 1 hr. Then highly

water-soluble tetrazolium salt, WST-8 was added to the cells and further incubated for 1 hour. Cell viability was assessed by reading the absorbance at 450nm.

Statistical analysis

Independent experiments were performed at least three times to allow statistical analysis. Normality was evaluated using the Shapiro–Wilk test and statistical significance was assessed by one-way ANOVA followed by Tukey’s post hoc test to analyze the difference between three or more groups. Dunnett's post hoc test was used for multiple comparisons using Graph Pad Prism. Results are presented as mean ± SEM and differences were considered statistically significant at p values ≤ 0.05.

Supplementary Figures and Legends

Supplementary Figure 1

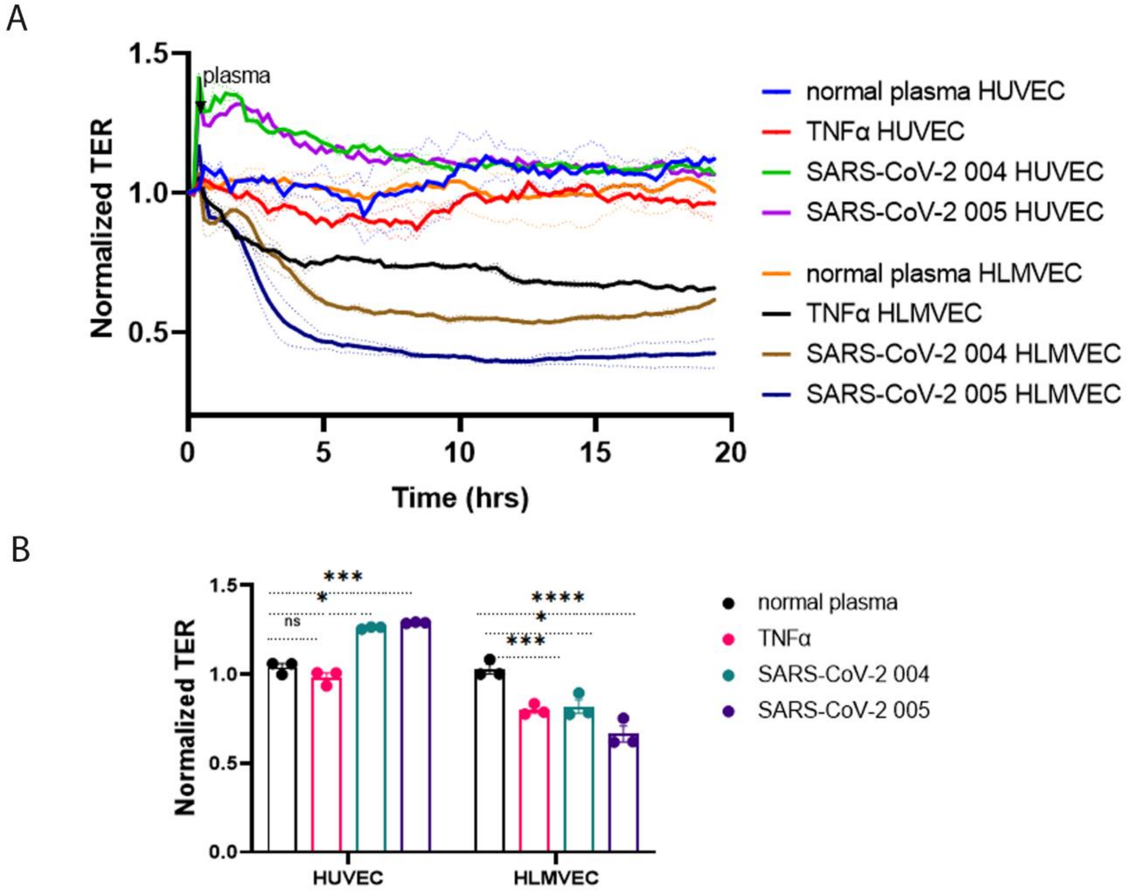

Figure S1. SARS-CoV-2 patient plasma does not alter endothelial permeability in human umbilical vein endothelial cells. A. HUVECs or HLMVEC were plated in ECIS arrays, a baseline was set, then cells were challenged either with normal or SARS-CoV-2 patient plasma (004 and 005) or with TNFα (1 ng/ml) and TER was monitored for 20 hours. Cells were treated at the 0-time point. B. Bar graph represents side by side resistance values of HUVEC and HLMVEC for normal plasma, SARS-CoV-2 plasma, and TNFα. Representative graph is shown from three independent experiments, n=3 in each group. *$p < 0.05$ normal plasma vs. SARS-CoV-2 004 in both HUVECs and HLMVECs, ***$p=0.0001$ normal plasma vs. SARS-CoV-2 005 in HUVECs

and normal plasma vs. TNFα in HLMVEC, ****p < 0.0001 normal plasma vs. SARS-CoV-2 005 in HLMVECs. The statistical significance was assessed by one-way ANOVA followed by Tukey's multiple comparisons post hoc test using Graph Pad Prism.

Supplementary Figure 2

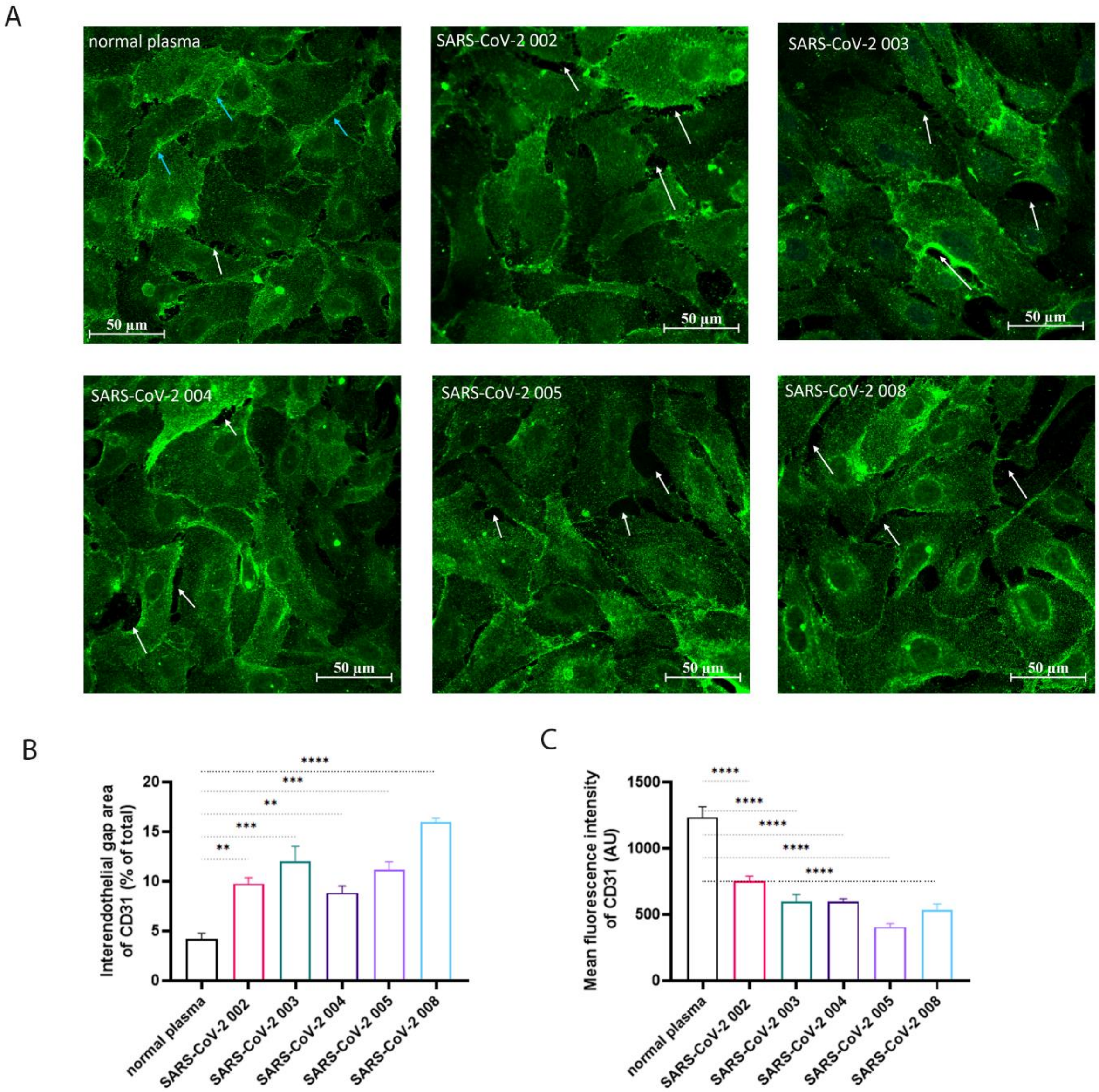

Figure S2. Impaired EC barrier after SARS-CoV-2 plasma treatment showed by CD31 staining. HLMVEC were treated either with normal pooled plasma (A) or with SARS-CoV-2 plasma in (1:200) 1 hour. Then cells were stained for CD31. Images were captured by Zeiss 780 Upright Confocal microscope, using 40x Plan-Apo (oil) objective. Scale bar is 50 µM. (B) Bar graph represents mean ±SEM of gap area on HLMVEC monolayers, expressed as a percent of the total area of each image treated either with normal or SARS-CoV-2 plasma (n=3). (C) Bar graphs represent mean ±SEM of fluorescence intensity of CD31 on HLMVEC monolayers treated either with normal or SARS-CoV-2 plasma (n=5). **$p < 0.01$, ***$p = 0.0001$, ****$p<0.0001$ vs. normal plasma. The statistical significance was assessed by one-way ANOVA followed by Tukey's multiple comparisons post hoc test using Graph Pad Prism.

Supplementary Figure 3

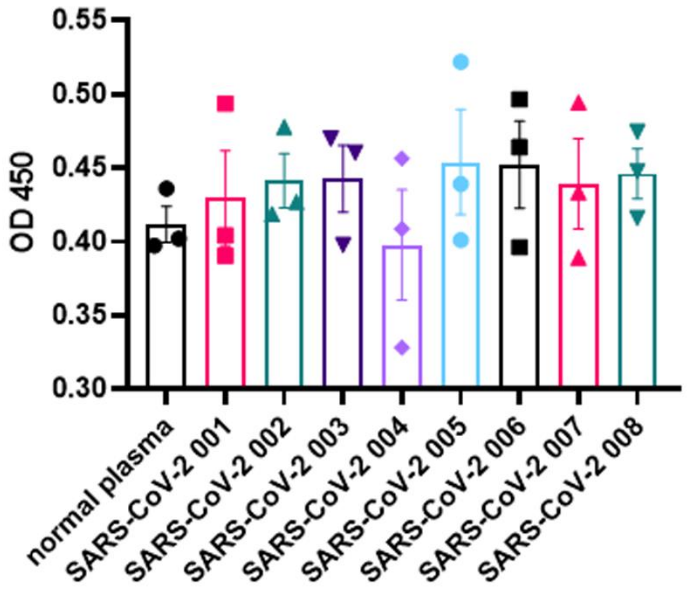

Figure S3. SARS-CoV-2 plasma has no effect on cell viability. HLMVEC were treated either with normal pooled plasma or with SARS-CoV-2 plasma in 1:200 dilution in normal growth media for 1 hour~~s~~. Water soluble tetrazolium (WST8) was added to the cells in cell survival proliferation assay at 1 hour~~s~~ after the plasma addition. Absorbance was measured at 450 nm. Representative data of 3 experiments .Values are present as Mean±SEM, n=8/group.

159

Supplementary Figure 4

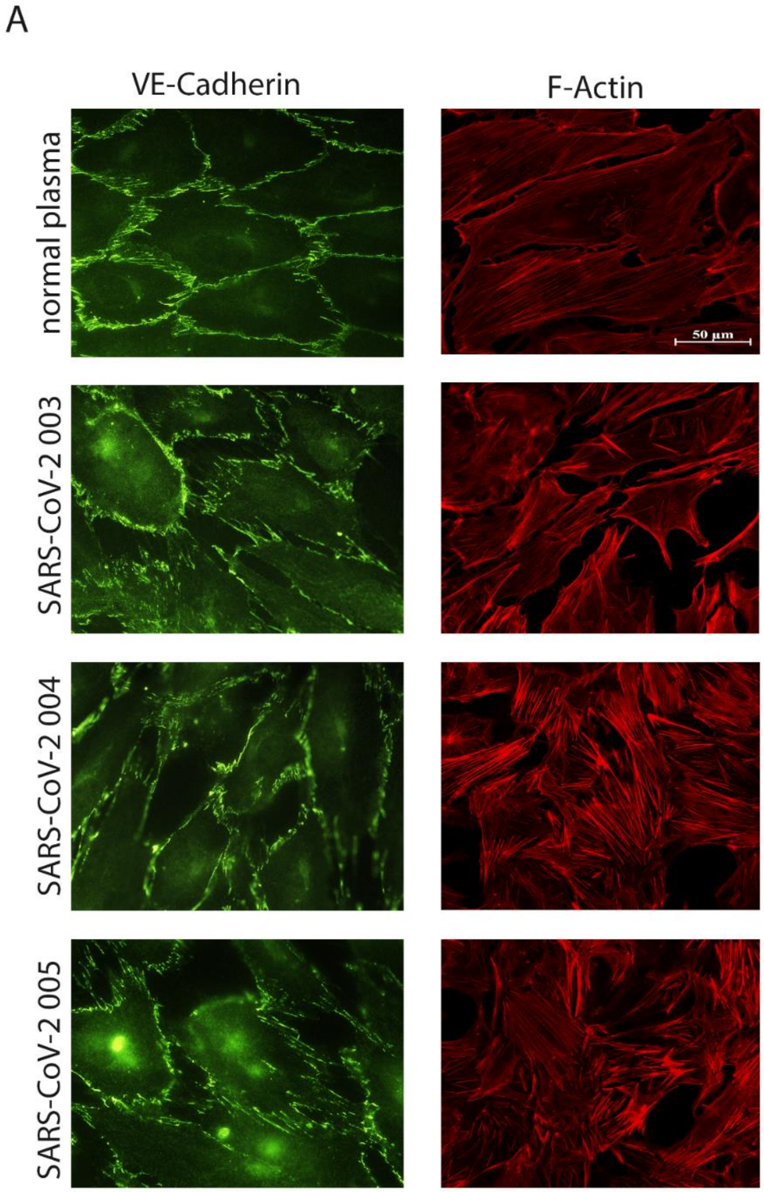

Supplementary Figure 4

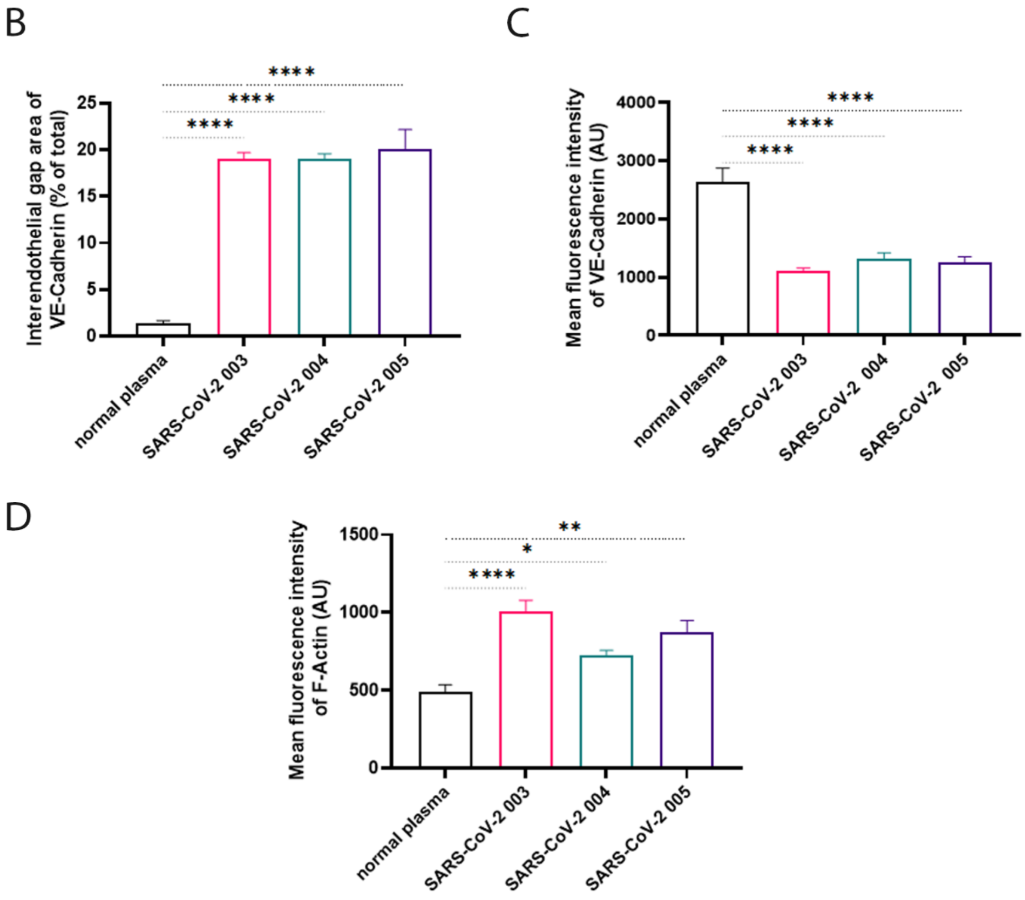

Figure S4. Disruption in Adherens Junctions and cytoskeletal rearrangement after SARS-CoV-2 plasma treatment. HLMVEC were treated either with normal pooled plasma (A) or with SARS-CoV-2 plasma in 1:200 dilution in normal growth media for 1 hour. Then cells were stained for VE-Cadherin or F-Actin by immunofluorescence. Images were captured by Zeiss 780 Upright Confocal microscope, using 40x Plan-Apo (oil) objective. (B) Bar graph represents mean ±SEM of gap area on HLMVEC monolayers, expressed as a percent of the total area of each image treated either with normal or SARS-CoV-2 plasma (n=3). (C) Bar graphs represent mean ±SEM of fluorescence intensity of VE-Cadherin on HLMVEC monolayers treated either with normal or SARS-CoV-2 plasma (n=5). (D) Bar graphs represent mean ±SEM of fluorescence intensity of F-

Actin on HLMVEC monolayers treated either with normal or SARS-CoV-2 plasma (n=5). Scale bar is 50 μM. Images were analyzed by the ZEN blue program. *$p < 0.05$, **$p < 0.01$, ****$p < 0.0001$ vs. normal plasma. The statistical significance was assessed by one-way ANOVA followed by Tukey's multiple comparisons post hoc test using Graph Pad Prism.

Supplementary Figure 5

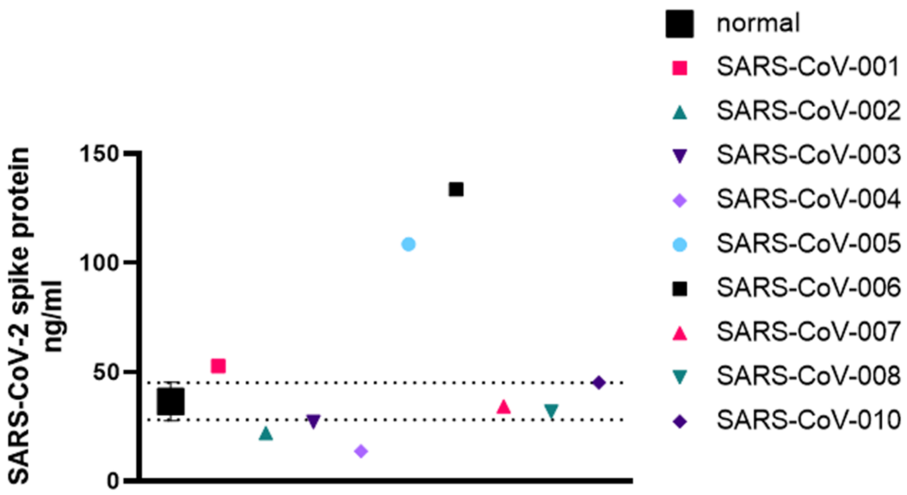

Figure S5. Spike protein concentration is not elevated in SARS-CoV-2 patients' samples. Spike protein concentrations were measured from SARS-CoV-2 patients' sera and compared to normal human serum (Sigma) using COVID-19 Spike Protein ELISA Kit (Abcam). OD values were measured at 450 nm. The dashed line shows the level of the SARS-CoV-2 spike protein in the normal plasma samples. Values are present as Mean±SEM.

Supplementary Figure 6

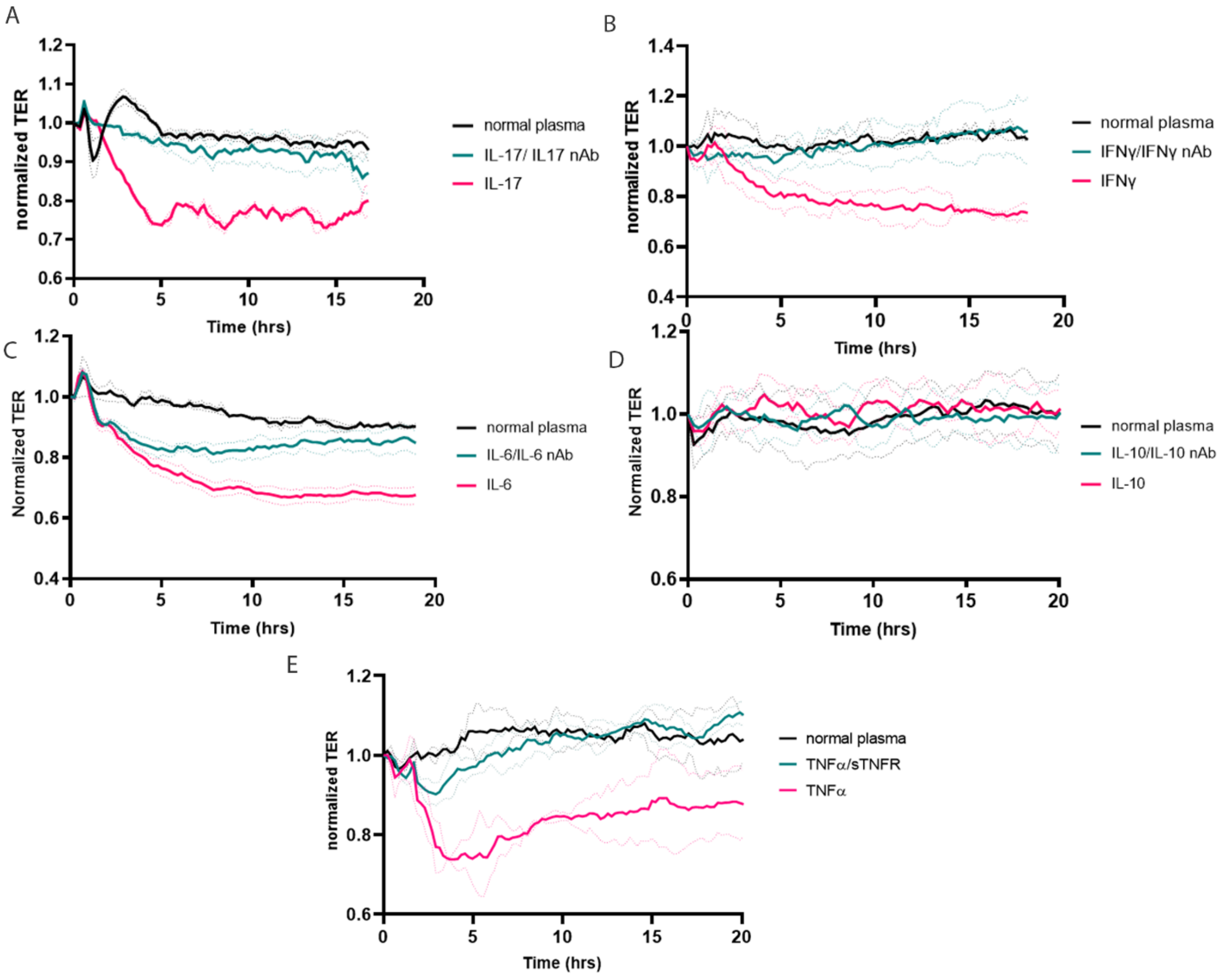

Figure S6. Effect of IL-17, IFNγ, IL-6, IL-10, and TNFα effect on endothelial permeability.

We tested the effect of A) IL-17 (1 ng/ml) with or without IL-17 neutralizing antibody (nAb) (5 ng/ml), B) IFNγ (1 ng/ml) with or without IFNγ nAb (1 ng/ml), C) IL-6 (20 ng/ml) with or without IL-6 nAb (5 ng/ml), and D) IL-10 (10 ng/ml) with or without IL-10 nAb (25 ng/ml) E) TNFα (1 ng/ml) with or without the soluble TNF receptor (1 ng/ml) on endothelial permeability. TER was monitored for 18-20 hours. Commercially available pooled normal human plasma was used as the normal plasma group control. Results are shown from 3 independent experiments, n=3.

Supplementary Figure 7

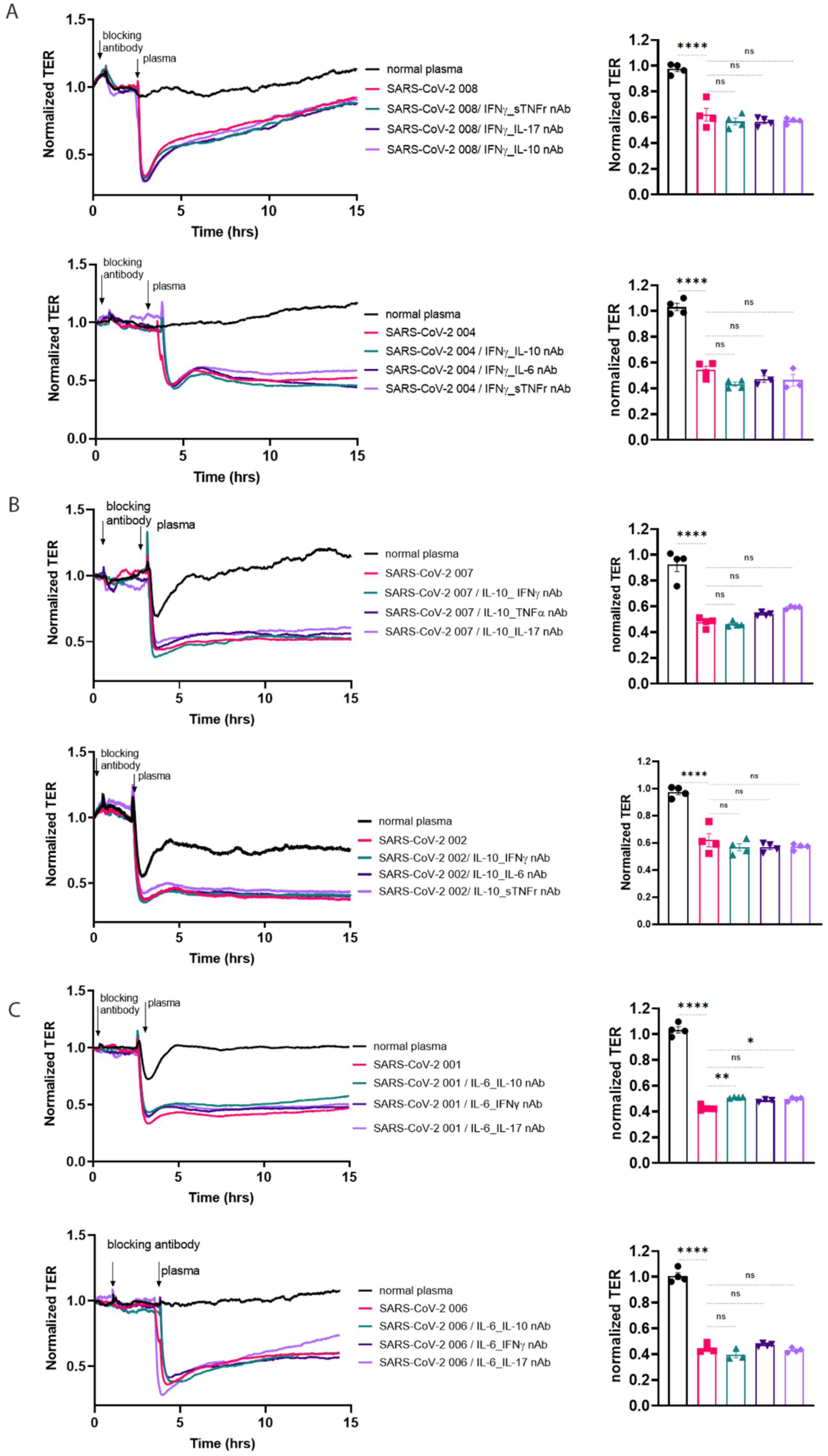

Supplementary Figure 7

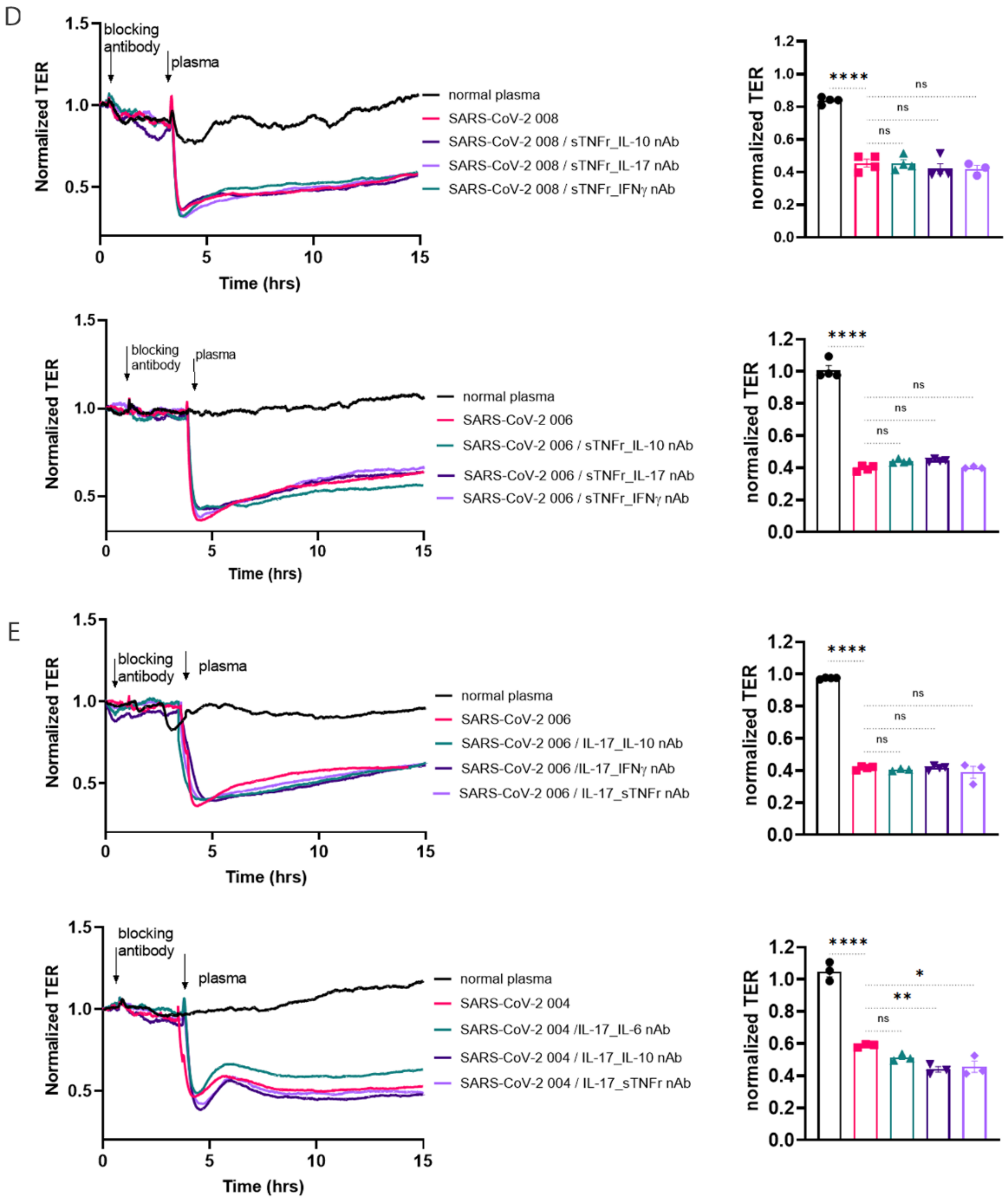

Figure S7. Combined blocking antibody combinations fail to reduce endothelial injury after exposure to SARS-CoV-2 plasma. HLMVECs monolayers were incubated with combinations of the indicated blocking antibodies for two hours prior to exposure to SARS-CoV-2 plasma (1:200 dilution in normal growth media) and TER was monitored for 18 hours. For each of the cytokines individually targeted in Figure 3 including IFNγ, IL-10, TNFα, IL-6, and IL-17. For each cytokine

target, 1 patient plasma with the second-highest fold increase and 1 patient plasma with the lowest or no increase was selected for the double targeting. (A) SARS-CoV-2 patient 008 and 004; B) SARS-CoV-2 patient 007 and 002; C) SARS-CoV-2 patient 001 and 006; D) SARS-CoV-2 patient 008 and 006; E) SARS-CoV-2 patient 006 and 004. For each of these selected patient plasma, the three other indicated cytokines with the highest increase were selected as the second target. Arrows indicate the treatment time points. Bar graphs represent the selected time points for statistical analysis to compare each treatment group at 2 hours after SARS-CoV-2 plasma treatment. Results are expressed as Mean ±SEM of 3 independent experiments, n=5. *$p < 0.05$, **$p < 0.01$, ****$p < 0.0001$ vs. normal plasma. The statistical significance was assessed by one-way ANOVA followed by Tukey's multiple comparisons post hoc test using Graph Pad Prism.

Supplementary Tables

Table S1. SARS-CoV-2 patient's characteristics.

SARS-CoV-2 patient	Sex	Age	Intubated day of admission	Intubated during clinical course	ICU Admission
**001**	F	69	N	Y	Y
**002**	F	78	N	N	N
**003**	M	41	N	Y	Y
**004**	F	70	N	N	N
**005**	F	76	N	N	Y
**006**	F	29	N	N	N
**007**	M	20	N	N	N
**008**	F	73	N	N	Y

Table S2. Pro-inflammatory cytokines involved in vascular permeability.

**cytokine**	**Levels in SARS-CoV-2 (total of 8 patients)**	**Effect on EC barrier**	**References**
IL-10	↑ in 8 patients	Inhibits vascular leakage	(6)
IL-6	↑ in 7 patients	Promotes loss of endothelial barrier function	(7, 8)
IFNγ	↑ in 7 patients	Increase vascular permeability	(9, 10)
TNFα	↑ in 6 patients	Disrupt EC barrier function	(11, 12)
IL-17A	↑ in 5 patients	Break down blood brain barrier	(13, 14)

Table S3. Neutralizing antibody combinations for each cytokine in 3 selected patients.

**Interferon γ**			
**SARS-CoV-2 patient**	**cytokine fold change**	**blocking antibody combinations**	**fold changes of 2nd cytokine target**
	increased	IFNγ / IL-10	25
SARS-CoV-2 006	3	IFNγ / IL-17	3
		IFNγ / sTNFr	1
	increased	IFNγ / sTNFr	2
SARS-CoV-2 008	3	IFNγ / IL-17	3
		IFNγ / IL-10	26
	no change	IFNγ / IL-10	21
SARS-CoV-2 004	0	IFNγ / IL-6	4
		IFNγ / sTNFr	2

Interleukin-10			
**SARS-CoV-2 patient**	**Cytokine fold change**	**blocking antibody combinations**	**fold changes of 2nd cytokine target**
	increased	IL-10 / IL-6	8
SARS-CoV-2 001	63	IL-10 / IFNγ	2
		IL-10 / IL-17	2
	increased	IL-10 / IFNγ	2
SARS-CoV-2 007	37	IL-10 / sTNFr	2
		IL-10 / IL-17	2
	increased	IL-10 / IL-6	7
SARS-CoV-2 002	11	IL-10 / IFNγ	2
		IL-10 / sTNFr	2

Tumor Necrosis Factor α			
**SARS-CoV-2 patient**	**cytokine fold change**	**blocking antibody combinations**	**fold changes of 2nd cytokine target**
	increased	sTNFr / IFNγ	3
SARS-CoV-2 008	2	sTNFr / IL-10	26
		sTNFr / IL-17	3
	increased	sTNFr / IL-10	22
SARS-CoV-2 005	2	sTNFr / IL-6	4
		sTNFr / IFNγ	1
	increased	sTNFr / IL-10	25
SARS-CoV-2 006	0.6	sTNFr / IL-17	4
		sTNFr / IL-IFNγ	6

**Interleukin-6**			
**SARS-CoV-2 patient**	**cytokine fold change**	**blocking antibody combinations**	**fold changes of 2nd cytokine target**
	increased	IL-6 / IL-10	63
SARS-CoV-2 001	8	IL-6 / IFNγ	2
		IL-6 / IL-17	2
	increased	IL-6 / IL-10	11
SARS-CoV-2 002	7	IL-6 / IFNγ	2
		IL-6 / sTNFr	2
	increased	IL-6 / IL-10	25
SARS-CoV-2 006	1	IL-6 / IFNγ	3
		IL-6 / IL-17	3

**Interleukin-17**			
**SARS-CoV-2 patient**	**cytokine fold change**	**blocking antibody combinations**	**fold changes of 2nd cytokine target**
	increased	IL-17 / sTNFr	2
SARS-CoV-2 008	3	IL-17 / IFNγ	3
		IL-17 / IL-10	26
	increased	IL-17 / IL-10	25
SARS-CoV-2 006	3	IL-17 / IFNγ	3
		IL-17 / sTNFr	1
	increased	IL-17 / IL-6	4
SARS-CoV-2 004	1	IL-17 / IL-10	21
		IL-17 / sTNFr	3

References

1. Findley CM, Mitchell RG, Duscha BD, Annex BH, Kontos CD. 2008. Plasma levels of soluble Tie2 and vascular endothelial growth factor distinguish critical limb ischemia from intermittent claudication in patients with peripheral arterial disease. J Am Coll Cardiol 52:387-93.
2. Ganta VC, Choi M, Farber CR, Annex BH. 2019. Antiangiogenic VEGF165b Regulates Macrophage Polarization via S100A8/S100A9 in Peripheral Artery Disease. Circulation 139:226-242.
3. Kovacs-Kasa A, Varn MN, Verin AD, Gonzales JN. 2017. Method for the Culture of Mouse Pulmonary Microvascular Endothelial Cells. Sci Pages Pulmonol 1:7-18.
4. Elshal MF, McCoy JP. 2006. Multiplex bead array assays: performance evaluation and comparison of sensitivity to ELISA. Methods 38:317-23.
5. Richens JL, Urbanowicz RA, Metcalf R, Corne J, O'Shea P, Fairclough L. 2010. Quantitative validation and comparison of multiplex cytokine kits. J Biomol Screen 15:562-8.
6. Li L, Elliott JF, Mosmann TR. 1994. IL-10 inhibits cytokine production, vascular leakage, and swelling during T helper 1 cell-induced delayed-type hypersensitivity. J Immunol 153:3967-78.
7. Alsaffar H, Martino N, Garrett JP, Adam AP. 2018. Interleukin-6 promotes a sustained loss of endothelial barrier function via Janus kinase-mediated STAT3 phosphorylation and de novo protein synthesis. Am J Physiol Cell Physiol 314:C589-C602.
8. Blecharz-Lang KG, Wagner J, Fries A, Nieminen-Kelha M, Rosner J, Schneider UC, Vajkoczy P. 2018. Interleukin 6-Mediated Endothelial Barrier Disturbances Can Be Attenuated by Blockade of the IL6 Receptor Expressed in Brain Microvascular Endothelial Cells. Transl Stroke Res 9:631-642.
9. Stewart RJ, Kashour TS, Marsden PA. 1996. Vascular endothelial platelet endothelial adhesion molecule-1 (PECAM-1) expression is decreased by TNF-alpha and IFN-gamma. Evidence for cytokine-induced destabilization of messenger ribonucleic acid transcripts in bovine endothelial cells. J Immunol 156:1221-8.
10. Youakim A, Ahdieh M. 1999. Interferon-gamma decreases barrier function in T84 cells by reducing ZO-1 levels and disrupting apical actin. Am J Physiol 276:G1279-88.
11. Clark PR, Kim RK, Pober JS, Kluger MS. 2015. Tumor necrosis factor disrupts claudin-5 endothelial tight junction barriers in two distinct NF-kappaB-dependent phases. PLoS One 10:e0120075.
12. Adam AP, Lowery AM, Martino N, Alsaffar H, Vincent PA. 2016. Src Family Kinases Modulate the Loss of Endothelial Barrier Function in Response to TNF-alpha: Crosstalk with p38 Signaling. PLoS One 11:e0161975.
13. Zenobia C, Hajishengallis G. 2015. Basic biology and role of interleukin-17 in immunity and inflammation. Periodontol 2000 69:142-59.
14. McGinley AM, Sutton CE, Edwards SC, Leane CM, DeCourcey J, Teijeiro A, Hamilton JA, Boon L, Djouder N, Mills KHG. 2020. Interleukin-17A Serves a Priming Role in Autoimmunity by Recruiting IL-1beta-Producing Myeloid Cells that Promote Pathogenic T Cells. Immunity 52:342-356 e6.
